# High Times for Painful Blues: The Endocannabinoid System in Pain-Depression Comorbidity

**DOI:** 10.1093/ijnp/pyv095

**Published:** 2015-09-05

**Authors:** Marie Fitzgibbon, David P. Finn, Michelle Roche

**Affiliations:** Physiology (Ms Fitzgibbon and Dr Roche), and Pharmacology and Therapeutics (Dr Finn), School of Medicine, Galway Neuroscience Centre and Centre for Pain Research (Ms Fitzgibbon, Dr Finn, and Dr Roche), National Centre for Biomedical Engineering Science, National University of IrelandGalway, Ireland.

**Keywords:** depression, pain, anandamide, cannabinoid, stress

## Abstract

Depression and pain are two of the most debilitating disorders worldwide and have an estimated cooccurrence of up to 80%. Comorbidity of these disorders is more difficult to treat, associated with significant disability and impaired health-related quality of life than either condition alone, resulting in enormous social and economic cost. Several neural substrates have been identified as potential mediators in the association between depression and pain, including neuroanatomical reorganization, monoamine and neurotrophin depletion, dysregulation of the hypothalamo-pituitary-adrenal axis, and neuroinflammation. However, the past decade has seen mounting evidence supporting a role for the endogenous cannabinoid (endocannabinoid) system in affective and nociceptive processing, and thus, alterations in this system may play a key role in reciprocal interactions between depression and pain. This review will provide an overview of the preclinical evidence supporting an interaction between depression and pain and the evidence supporting a role for the endocannabinoid system in this interaction.

## Clinical Data Supporting Depression-Pain Comorbidity

Depression and pain are two of the most prevalent psychiatric and neurological disorders worldwide, and both are associated with significant disability, impaired health-related quality of life, and high mortality (Spitzer et al., 1995; Kvien, 2004; Scholich et al., 2012; Hassett et al., 2014). While each is considered a debilitating disorder in its own right, these disease entities frequently coexist, and it has been reported that this association may be as high as 80% of patients (Poole et al., 2009). For example, major depressive and bipolar disorder is associated with painful symptoms in up to 95% of patients (Grover et al., 2012; Maneeton et al., 2013; Nicholl et al., 2014). Similarly, patients suffering from inflammatory and neuropathic pain are up to 4.9 times more likely to develop depression or anxiety disorder than the general population (Hawker et al., 2011; Knaster et al., 2012; Emery et al., 2014; Lin et al., 2015). Patients exhibiting comorbid depression and pain do not respond as effectively to pharmacological treatment, and this comorbidity is more disabling and expensive to both patients and society than either condition alone (Emptage et al., 2005; Gameroff and Olfson, 2006). Furthermore, it has also been found that the severity of depression directly correlates with increased severity of pain symptomatology (Khongsaengdao et al., 2000). However, it should be noted that an intricate relationship exists between depression and pain such that although pain is commonly reported by depressed patients, examination of pain thresholds to various stimuli such as cold, heat, and pressure have been shown to be reduced, increased, or unchanged (Ben-Tovim and Schwartz, 1981; Lautenbacher et al., 1999; Gormsen et al., 2004; Bar et al., 2005; Boettger et al., 2013), effects which depend on the modality and intensity of the stimulus. Thus, given the complex interaction between affect and pain, and the high comorbidity of depression-pain, greater understanding of the neurobiological mechanisms underlying the association is warranted to develop more efficacious treatment strategies. There have been several studies investigating the role of neural substrates, including neuroanatomical organization, neurotransmission, neurotrophins, dysregulation of the hypothalamo-pituitary-adrenal (HPA) axis, and inflammation, to name but a few, in the interaction between affect and nociceptive processing (for review, see Blackburn-Munro, 2004; Goesling et al., 2013; Walker et al., 2014). A full review of the role of each of these substrates is beyond the scope of this review. However, increased evidence has indicated a role for a further substrate, the endogenous cannabinoid (endocannabinoid) system, in affective and nociceptive responding (for reviews, see Finn, 2010; Ashton and Moore, 2011; Gorzalka and Hill, 2011; Rani Sagar et al., 2012; Hillard and Liu, 2014; Jennings et al., 2014; Boychuk et al., 2015) and as such, alterations in this system may provide a common mechanism by which depression and pain coexist. Preclinical animal models provide a valuable means of investigating potential neurobiological substrates that may underlie the association between depression and pain. As such, this review will provide an overview of the preclinical evidence supporting an interaction between depression and pain, the evidence supporting a role for the endocannabinoid system in this interaction, and the potential mechanisms through which the endocannabinoid system may mediate effects on affect and nociceptive processing.

## Preclinical Animal Models Support Depression-Pain Interactions

### Animal Models of Depression Exhibit Altered Nociceptive Responding

Several animal models of depression based on genetics, stress, lesion, and pharmacological manipulation have been shown to exhibit alterations in nociceptive responding (for review, see Li, 2015), supporting the clinical finding of an association between depression and pain. For example, in rats, the chronic mild stress model of depression has been shown to display a reduced nociceptive threshold to cold (Bardin et al., 2009; Bravo et al., 2012; Bravo et al., 2014) and mechanical (Bardin et al., 2009; Imbe et al., 2012) stimuli and an increased threshold to noxious thermal stimuli (Shi et al., 2010). Furthermore, both inflammatory (Gameiro et al., 2005; Rivat et al., 2010; Wang et al., 2013) and neuropathic (Bravo et al., 2012) pain behavior are enhanced in chronic stress models of depression. Similarly, we and others have shown that the Wistar-Kyoto (WKY) rat, a stress hyperresponsive rat strain with a depressive-like phenotype, exhibits thermal hyperalgesia (Burke et al., 2010), visceral hyperalgesia to colorectal distension (Gibney et al., 2010; Gosselin et al., 2010; O’Malley et al., 2010), enhanced formalin-evoked inflammatory pain behavior (Burke et al., 2010; Rea et al., 2014), and enhanced mechanical allodynia following peripheral nerve injury (neuropathic pain) (Zeng et al., 2008; del Rey et al., 2011). Reserpine-induced monoamine depletion has long been known to result in depressive-like behavior, and recent evidence has demonstrated accompanying thermal allodynia (Liu et al., 2014), as well as pronounced and long-lasting mechanical hyperalgesia and allodynia, and cold allodynia (Nagakura et al., 2009; Arora et al., 2011). Thus, this model has been proposed as a possible rodent model of fibromyalgia (Nagakura et al., 2009). Furthermore, recent work from our group has demonstrated that the olfactory bulbectomised rat, a lesion model of depression, exhibits increased sensitivity to mechanical and thermal stimuli in the von Frey, acetone drop, hot plate, and tail flick tests (Burke et al., 2010, 2013), increased inflammatory pain responding in the formalin test (Burke et al., 2010), and enhanced neuropathic pain responding following spinal nerve ligation (Burke et al., 2013, 2014). Thus, taken together, several animal models of depression have been shown to exhibit altered nociceptive thresholds and enhanced inflammatory and neuropathic pain behavior, mimicking effects observed clinically.

### Animal Models of Chronic Pain Exhibit Depressive-Like Behavior

Depressive- and anxiety-like behavior, as assessed by multiple paradigms, has been reported in a wide variety of preclinical models of chronic pain (for review, see Yalcin et al., 2014; Li, 2015). For example, peripheral or spared nerve injury in mice induces a pronounced mechanical allodynia accompanied by the development of depressive-like behavior as determined by enhanced immobility in the forced swim test (FST) (Goncalves et al., 2008; Norman et al., 2010; Wang et al., 2011). Similarly, rodents subjected to the chronic constriction injury model of neuropathic pain exhibit reduced sucrose preference (Dellarole et al., 2014) and increased immobility in the FST (Hu et al., 2009; Jesse et al., 2010; Fukuhara et al., 2012; Zhao et al., 2014), indicating the development of anhedonia and behavioral despair, hallmarks of depressive-like behavior. In the complete Freund’s adjuvant model of inflammatory pain, both mice and rats exhibited depression-like behavior in the FST (Maciel et al., 2013; Borges et al., 2014) and tail suspension test (Maciel et al., 2013) and anxiety-related behavior in the elevated plus maze, open field test, and social interaction test (Parent et al., 2012; Borges et al., 2014). Such changes in affective processing in chronic pain models have been shown to occur later than the development of enhanced somatosensory perception. For example, in neuropathic pain models, alterations in emotional behavior have been observed 4 to 8 weeks post nerve injury (Suzuki et al., 2007; Yalcin et al., 2011), but not prior to this (2-4weeks) when mechanical allodynia/hypersensitivity is observed (Kontinen et al., 1999; Hasnie et al., 2007). These studies highlight the development of depressive- and anxiety-like behavior in models of neuropathic or inflammatory pain and suggest that pathological alterations induced by persistent nociceptive input to brain regions that process both pain and affect may account, at least in part, for comorbid depressive-like behavioral changes.

Animal models that replicate the clinical scenario are important for in-depth investigation of the possible neurobiological substrates that may mediate the association between depression and chronic pain. Although the cause of this coexistence remains somewhat elusive, as highlighted earlier, common anatomical substrates and neurobiological mediators, including neurotransmitters, neurotrophins, neuroendocrine alterations, and inflammatory mediators, have been identified, any or all of which may alter neural functioning in key brain regions involved in regulating emotional and nociceptive processing (for review, see Blackburn-Munro, 2004; Maletic and Raison, 2009; Anderson et al., 2012; Goesling et al., 2013; Meerwijk et al., 2013; Jennings et al., 2014; Walker et al., 2014; Doan et al., 2015). Increasing evidence has highlighted an important role for the endocannabinoid system in modulating emotional and nociceptive processing, and thus this system may play a key role in the association between pain and depression.

### The Endocannabinoid System

The plant *Cannabis sativa* has been used as a medicine throughout the world for several thousand years, with reports of its use in treating painful symptoms appearing as early as 2600 bc. The principal psychoactive ingredient of *Cannabis sativa*, delta-9-tetrahydrocannabinol (Δ^9^-THC), was first identified in 1964 (Gaoni and Mechoulam, 1964), and subsequent studies to understand its mechanism of action led to the discovery of the endogenous cannabinoid (endocannabinoid) system. This endocannabinoid system consists of the cannabinoid receptors (CB_1_ and CB_2_) (Devane et al., 1988; Matsuda et al., 1990; Munro et al., 1993), their naturally occurring endogenous ligands (the best characterized of which are N-arachidonoylethanolamine, or anandamide [AEA]) (Devane et al., 1992) and 2-arachidonylglycerol (2-AG) (Mechoulam et al., 1995), and the enzymes involved in their biosynthesis and degradation. Other endocannabinoid ligands have also been identified, including oleamide (Leggett et al., 2004), O-arachidonoyl ethanolamine (virodhamine) (Porter et al., 2002), 2-arachidonoyl glycerol ether (noladin ether) (Hanus et al., 2001), and N-arachidonoyl-dopamine (Huang et al., 2002), although their physiological role has not been examined in detail. Endocannabinoid biosynthesis occurs on demand via hydrolysis of cell membrane phospholipid precursors. AEA is formed from the precursor *N*-arachidonoylphosphatidylethanolamine due to the hydrolytic activity of the phospholipase D enzyme NAPE-PLD (Di Marzo et al., 1994; Sugiura et al., 1996), while fatty acid amide hydrolase (FAAH) is the primary enzyme responsible for the metabolism of this endocannabinoid (Cravatt et al., 1996). In comparison, the main biosynthetic pathway for 2-AG involves the hydrolysis of the membrane phospholipid phosphatidylinositol by phospholipase C, producing 1,2-diacylglycerol, which is then converted to 2-AG by diacylglycerol lipase (Prescott and Majerus, 1983, Sugiura et al., 1995). 2-AG is primarily metabolized by monoacylglycerol lipase (MAGL) (85%) (Blankman et al., 2007), although other enzymes including cyclooxygenase-2 (Yu et al., 1997; Kozak et al., 2000), lipoxygenase (van der Stelt et al., 2002), ABDH6 (serine hydrolase α/β-hydrolase domain), and ABDH12 (Blankman et al., 2007), have also been shown to play a role.

Upon release, endocannabinoids bind and activate the G-protein coupled receptors CB_1_ and/or CB_2_. CB_1_ receptors are highly expressed on presynaptic neurons throughout the human and rodent brain (Herkenham, 1991; Tsou et al., 1998; Mackie, 2008), the activation of which results in inhibition of cyclic AMP, activation of mitogen-activated protein kinase, inhibition of N- and P/Q-type voltage-activated Ca^2+^ channels, and induction of inwardly rectifying K+ currents, with the resultant inhibition of neurotransmitter release (Demuth and Molleman, 2006). CB_1_ receptors have also been shown to be expressed on glia and a wide range of peripheral tissues, though at lower levels than observed on neurons (Galiegue et al., 1995; Carlisle et al., 2002; Osei-Hyiaman et al., 2005; Cavuoto et al., 2007; Cota, 2007). In contrast, CB_2_ receptors are widely distributed in peripheral tissues and organs, with a particularly high density on immune cells and tissues (Munro et al., 1993; Berdyshev, 2000; Sugiura et al., 2000), including on glia within the brain, with enhanced expression observed under neuroinflammatory conditions (Carlisle et al., 2002; Nunez et al., 2004; Rock et al., 2007). Accumulating evidence has also indicated that the CB_2_ receptor is also expressed on subsets of neurons within the brain (Van Sickle et al., 2005; Gong et al., 2006; Baek et al., 2008; Zhang et al., 2014) and thus also modulates neurotransmission (Roche and Finn, 2010; Atwood et al., 2012; Kim and Li, 2015). Endocannabinoids also have affinity for and activity at other receptors, namely the transient receptor potential vanilloid 1, peroxisome proliferator-activated receptors, GPR55, and GPR119 (Huang et al., 2002; Overton et al., 2006; Sun et al., 2006; Ryberg et al., 2007). Activity at these receptors has been proposed to account, at least partially, for some of the differential effects observed with potent selective cannabinoid agonists and pharmacological modulators of endocannabinoid tone.

Because of the distribution of the endocannabinoid system throughout spinal and supraspinal regions, it is in a prime position to regulate neurophysiological activities such as affective and nociceptive processing. This has been a very active area of research over the past decade, with a number of excellent reviews synthesizing the data supporting a role for the endocannabinoid system in modulating mood and nociception (for review, see Ashton and Moore, 2011; Gorzalka and Hill, 2011; Rani Sagar et al., 2012; Hillard and Liu, 2014; Ulugol, 2014; Boychuk et al., 2015). However, no review to date has examined the evidence that may support a role for the endocannabinoid system as a link between depression and pain, and thus the remainder of this review aims to collate and synthesize these data.

## The Role of the Endocannabinoid System In Depression-Pain Interactions

### Clinical Evidence

Several lines of evidence have demonstrated alterations in the endocannabinoid system in chronic pain (Richardson et al., 2008; Kaufmann et al., 2009) and in psychiatric patients (Gobbi et al., 2005; Hill and Gorzalka, 2005; Koethe et al., 2007). For example, various polymorphisms of CB_1_ and CB_2_ receptors have been identified in patients with major depression and bipolar disorder (Monteleone et al., 2010; Minocci et al., 2011; Mitjans et al., 2012; Mitjans et al., 2013) with a single nucleotide polymorphism in the CB_1_ receptor reported to enhance the risk of treatment resistance in depression (Domschke et al., 2008) and the development of anhedonic depression following early life trauma (Agrawal et al., 2012). Similarly, genetic alterations in the CB_1_ receptor and FAAH have also been identified in patients with pain associated with migraine, Parkinson’s disease, and irritable bowel syndrome (Juhasz et al., 2009; Park et al., 2011; Greenbaum et al., 2012). In addition, serum levels of endocannabinoids have been reported to be reduced in both depressed patients (Hill et al., 2008c, 2009b) and chronic pain patients (Fichna et al., 2013). A recent study has reported enhanced plasma 2-AG levels and increased CB_1_ and CB_2_ mRNA expression on blood lymphocytes in osteoarthritic patients (La Porta et al., 2015). A significant positive correlation was observed between 2-AG levels, pain, and depression, and a negative correlation of 2-AG with quality of life and visual memory was observed (La Porta et al., 2015). In addition, CB_1_ receptor expression was positively correlated with depression scores, while CB_2_ receptor expression was correlated with pain scores. These data indicate that key components of the endocannabinoid system are upregulated in human osteoarthritis with significant correlations with pain and emotional symptoms. In addition to visual loss and sensory deficits, neuromyelitis optica is associated with significant pain (altered threshold responding and symptoms of neuropathic pain), depression, and increased plasma levels of 2-AG and AEA (Pellkofer et al., 2013). This study evaluated if a correlation existed between pain threshold and levels of endocannabinoids, demonstrating a considerable negative correlation between the plasma levels of 2-AG and mechanical pain thresholds in these patients, although this study did not evaluate if an association also existed with depressed mood. While the data suggest a possible association between pain, depression, and the endocannabinoid system in osteoarthritis and neuromyelitis optica patients, further clinical studies are required to determine if alterations in the genetics, levels, and activity of the endocannabinoid system exist in other patient groups exhibiting depression-pain comorbidity.

There has been a paucity of clinical studies directly investigating the role or activity of cannabinoids in depression-pain interactions; however, enhanced mood and improved quality of life have been reported in studies investigating the analgesic efficacy of cannabinoid-based therapies (Table 1). For instance, cannabis intake has been reported to improve muscle and nerve pain as well as depression and anxiety symptomatology in a group of HIV patients (Woolridge et al., 2005). Improvements in anxiety and overall distress have been reported in patients with advanced cancer in whom pain symptoms were managed by daily adjunctive administration of Cesamet (nabilone, a Δ^9^-THC analogue) for 30 days (Maida et al., 2008). Similarly, a randomized, double blind, placebo-controlled trial, which examined the therapeutic benefit of nabilone in terms of pain management and quality of life improvement in patients with fibromyalgia, identified significant pain relief and alleviation of anxiety symptoms after 4 weeks of therapy (Skrabek et al., 2008). In addition, a retrospective evaluation investigating the efficacy of nabilone for the management of concurrent disorders in seriously mentally ill correctional populations identified significant amelioration of symptoms related to posttraumatic stress disorder as well as a subjective improvement in chronic pain (Cameron et al., 2014). Furthermore, a multicenter retrospective survey of patients with chronic central neuropathic pain or fibromyalgia who were prescribed oral Δ^9^-THC (dronabinol), supplemental to existing medication, reported improved symptoms of both anxiety and depression after 7 months of treatment as assessed by the Hospital Anxiety and Depression Scale (Weber et al., 2009). Although Sativex (1:1 ratio of Δ^9^-THC:cannabidiol), indicated for resistant spasticity and pain in multiple sclerosis, has not yet been directly associated with significant mood changes, patients have reported improvements in overall quality of life following 16 weeks of treatment (Vermersch, 2011). In a separate randomized control clinical trial evaluating the effect of Sativex in patients with chronic painful diabetic neuropathy, patients with comorbid depression displayed significant improvements in total pain score in comparison with nondepressed counterparts (Selvarajah et al., 2010). Collectively, the above studies suggest that when coexistent, both depression/anxiety and pain respond to exogenously administered cannabinoids, although it remains to be determined if the effects are mediated by common or parallel mechanisms. Recent evidence has demonstrated enhanced amygdala activity and reduced functional connectivity between the amygdala and somatosensory cortex correlate with Δ^9^-THC–mediated reductions in the unpleasantness to ongoing pain (Lee et al., 2013), suggesting that the amygdala may provide a common neural circuit for the association between emotional responding and pain.

**Table 1.** Clinical Studies Demonstrating Effects of Cannabinoid-Based Therapies on Symptoms of Comorbid Depression and Pain

	Drug	Pain Measurement	Depression/ Anxiety Measurement	Outcomes in Pain	Outcomes in Depression/ Anxiety	Reference
HIV	Cannabis	Pilot questionnaire	Pilot Questionnaire	muscle, ↓ nerve pain, headaches	↓ anxiety, depression	Woolridge et al. (2005)
Cancer pain	Nabilone (Cesamet®)	ESAS MSE	ESAS	↓ pain score, MSE	↓ anxiety, overall stress	Maida et al. (2008)
Fibromyalgia	Nabilone	VAS FIQ	Anxiety	↓ pain	↓ anxiety	Skrabek et al. (2008)
Mentally ill offenders	Nabilone	Self-reported pain severity	PCL-C GAF	↓ pain	↓ PTSD symptoms	Cameron et al. (2014)
Multiple sclerosis- related resistant spasticity	Sativex (Δ^9^-THC, cannabidiol)	NRS spasticity score	QOL	↓ spasticity	↑ QOL	Vermersch (2011)
Chronic central neuropathic pain, fibromyalgia	Δ^9^-THC	VRS, NRS, PDI	SF-12, QLIP, HADS,	↓ pain, pain intensity	↑ QOL, depression, ↓ anxiety	Weber et al. (2009)
Painful diabetic peripheral neuropathy	Sativex (Δ^9^-THC, cannabidiol)	VAS	HADS, QOL	↓ pain (only in patients with baseline depression)	↑ QOL	Selvarajah et al. (2010)

Abbreviations: ESAS, Edmonton symptom assessment system; FIQ, fibromyalgia impact questionnaire; GAF, Global Assessment of Functioning; HADS, hospital anxiety and depression scale; HIV, human immunodeficiency virus; MSE, morphine sulphate equivalent; NRS, numerical rating scale; PDI, pain disability index; PCL-C, Posttraumatic Checklist–Civilian version; PTSD, posttraumatic stress disorder; QLIP, quality of life; QOL, quality of life; SF-12, short form-12;VAS, visual analog scale; VRS, verbal rating scale.

### Preclinical Evidence

Despite numerous reports of altered endocannabinoid signaling in various animal models of pain (Lim et al., 2003; Zhang et al., 2003; Walczak et al., 2005; Mitrirattanakul et al., 2006) and mood-related behavior (Vinod et al., 2012; Marco et al., 2014; Navarria et al., 2014), there is limited direct evidence available identifying alterations in endocannabinoid function in animal models of coexistent depressive and pain behavior (Tables 2 and 3). One of the first studies examining the role of the endocannabinoid system in the interaction between affect and pain was conducted by Takahashi and colleagues (2003). In this study, outbred Swiss-albino mice were stratified into groups of anxious and nonanxious animals as determined by behavioral responses in the elevated plus maze, before subsequent exposure to intraplantar formalin administration (Takahashi et al., 2003). Despite the hypothesis that the degree of anxiety may contribute to the perception of and response to the noxious stimulus, both anxious and nonanxious animals displayed comparable formalin-evoked biphasic nociceptive profiles. Systemic pretreatment with Δ^9^-THC elicited an analgesic effect in both groups of animals, an effect blocked by systemic pretreatment with a CB_1_ receptor antagonist, rimonabant (Takahashi et al., 2003). These data suggested that the endocannabinoid system (and in particular the CB_1_ receptor) may represent a potential treatment strategy for inflammatory pain in the presence and/or absence of anxiety and possibly other neuropsychiatric disorders. However, it should be noted that direct activation of central CB_1_ receptors is responsible for the psychoactive effects of potent synthetic or plant-derived cannabinoids; thus potent, direct agonism of this receptor is unlikely to be therapeutically viable for pain and/or psychiatric disorders. In comparison, modulation of endocannabinoid tone by inhibiting enzymes responsible for their metabolism has been proposed to confer improved efficacy and safety relative to direct cannabinoid agonists.

**Table 2.** Endocannabinoid-Mediated Effects/Changes on Affective and Nociceptive Behavior in Animal Models

Depression/ Affective Model	Nociceptive Effects	Cannabinoid-based drugs		Endocannabinoid-related changes/effects	Reference
Anxiety-stratified (EPM), mouse	↑ formalin-evoked nociceptive responding in anxious and non- anxious	Δ^9^-THC Rimonabant	CB_1/2_ agonist CB_1_ antagonist	Δ^9^-THC ↓ nociception in both anxious and non-anxious mice, Rimonabant blocked effects of Δ^9^-THC	Takahashdi et al. (2003)
WKY rat	↑ formalin-evoked nociceptive responding	URB597 AM251	FAAH inhibitor CB_1_ antagonist	Formalin-induced ↓ AEA in RVM, No formalin-induced ↑ 2-AG, NAPE-PLD or DAGL-α in RVM (compared with SD) Systemic URB597 ↓ nociception Systemic AM251 ↑ nociception AM251 within RVM blocked effect of URB597	Rea et al. (2014)
Repeated FST in SD and WKY rat	Stress ↓ formalin- evoked nociceptive responding in SD Stress ↓ formalin- evoked nociceptive responding in WKY			↑ MAGL mRNA in spinal cord of SD ↓ AEA in amygdala of SD No change in MAGL mRNA in spinal cord of WKY No change AEA in amygdala of WKY	Jennings et al. (2015)
CUS, mouse	↓ latency to respond in HPT	URB597 JZL184	FAAH inhibitor, MAGL inhibitor	URB597 ↓ anxiety (EPM, LD) JZL184 ↓ anxiety (LD) Both ↓ thermal hyperalgesia	Lomazzo et al. (2015)
CUS, mouse	Chronic mechanical hyperalgesia following NGF	URB597 JZL184	FAAH inhibitor, MAGL inhibitor	URB597 ↓ hyperalgesia No change with JZL184	Lomazzo et al. (2015)

Abbreviations: AEA, anandamide; 2-AG, 2-arachidonoylglycerol; CUS, chronic unpredicted stress; EPM, elevated plus maze; FAAH, fatty acid amino hydrolase; DAGL-α, diacylglycerol lipase-alpha; HPT, hot plate test; LD, light-dark box; MAGL, monoacylglycerol lipase; NAPE-PLD, N-acyl phosphatidylethanolamine-specific phospholipase D; NGF, nerve growth factor; RVM, rostral ventromedial medulla; SD, Sprague Dawley; WKY, Wistar-Kyoto.

**Table 3.** Endocannabinoid-Mediated Effects/Changes on Affective and Nociceptive Behavior in Animal Models of Pain

Pain Model	Depressive Effects	Cannabinoid-Based Drugs		Endocannabinoid-Related Changes/Effects	Reference
PNL, mouse	↑ Anxiety in LD and Zero Maze ↓ Sucrose Preference in CB_1_^-/-^ mice only			Anxiety and depressive effects only in CB_1_^-/-^ mice	Racz et al. (2015)
Monosodium iodoacetate, mouse	↑ Anxiety in EPM Memory impairment in object recognition memory task	ACEA JWH133	CB_1_ agonist CB_2_ agonist	↑ anxiety in CB_1_^-/-^ mice no anxiety in CB_2_^-/-^ mice ACEA and JWH133 ↓ mechanical allodynia and anxiety ACEA ↓ memory impairment	La Porta et al. (2015)
CCI, rat	↑ Immobility in FST	GW405833	CB_2_ agonist	GW405833 ↓ mechanical hyperalgesia GW405833 ↓ immobility	Hu et al. (2009)
Acid-stimulated stretching, rat	↓ Food intake ↓ ICSS	Δ^9^-THC, CP55940	CB_1/2_ agonist CB_1/2_ agonist	Both blocked stretching Both exacerbated ↓ ICSS No effect on feeding	Kwilacz et al. (2012)
Acid-stimulated stretching, rat	↓ ICSS	URB597 Rimonabant SR144528	FAAH inhibitor CB_1_ antagonist CB_2_ antagonist	URB597 ↓ stretching; blocked by rimonabant, URB597 induced delayed partial attenuation of ICSS - not attenuated by rimonabant or SR144528	Kwilacz et al. (2014)

Abbreviations: CCI, chronic constrictive injury; CFA, complete Freud’s adjuvant; EPM, elevated plus maze; FAAH, fatty acid amino hydrolase; FST, forced swim test; ICSS, intracranial self-stimulation; LD, light-dark box; MBT, marble burying test; PNL, partial sciatic nerve ligation.

The WKY rat is a genetically stress-sensitive strain of rat that exhibits a depression- and anxiety-related phenotype (Pare and Redei, 1993) and heightened nociceptive behavioral responding in several paradigms (Zeng et al., 2008; Burke et al., 2010). Characterization of the endocannabinoid system in WKY rats has revealed higher levels of FAAH and CB_1_ receptor coupling and lower levels of AEA in the frontal cortex and hippocampus when compared with Wistar rats (Vinod et al., 2012). Furthermore, enhancing AEA tone by pharmacologically inhibiting FAAH activity resulted in an attenuation of depressive-like behavior (sucrose preference test and FST) in WKY rats (Vinod et al., 2012). Recent studies in our laboratory have identified alterations in the endocannabinoid system concurrent with enhanced formalin-evoked nociceptive behavior in the WKY rat (Rea et al., 2014) (Table 2). More specifically, we found that in WKY rats, intraplantar administration of the noxious inflammatory pain stimulus formalin resulted in a significant reduction in AEA in the rostral ventromedial medulla (RVM), a component of the descending pain pathway synonymous with pain facilitation and/or inhibition, an effect that was not observed in Sprague Dawley (SD) counterparts. Intraplantar administration of formalin increased levels of 2-AG in the RVM of SD rats, an effect not observed in WKY animals. Furthermore, exposure to formalin induced significant increases in mRNA expression of NAPE-PLD and diacylglycerol lipase-α, precursors of AEA and 2-AG, respectively, in the RVM of SD rats, an effect not observed in the WKY strain. Pharmacological studies were carried out to evaluate the functional significance of the alterations in the endocannabinoid system in WKY rats in response to formalin. Enhancing endogenous AEA tone following systemic administration of the FAAH inhibitor URB597 attenuated formalin-evoked hyperalgesic responding in WKY rats, while in comparison, CB_1_ receptor antagonism was associated with augmentation of nociceptive responding. Furthermore, CB_1_ receptor blockade within the RVM attenuated the reduction in nociceptive behavior induced by URB597 in WKY rats. In comparison, pharmacological manipulation of the endocannabinoid system in SD rats did not alter formalin-evoked nociceptive responding (Rea et al., 2014). These findings indicate a causative role of endocannabinoid dysregulation in hyperalgesic behavior associated with negative affect, and moreover identify a role for AEA-induced activation of CB_1_ receptors in the RVM as a mediator of pain suppression in animal subjects predisposed to anxiety and depression. In addition to its role in influencing responsivity of WKY rats to noxious stimuli in the absence of stress, we have also shown very recently that the endocannabinoid system may also play a role in the differential effects of repeated homotypic stress on inflammatory pain-related behavior in WKY vs SD rats (Jennings et al., 2015). Specifically, repeated forced swim stress exposure prolonged and attenuated formalin-evoked nociceptive behavior in SD and WKY rats, respectively. These behavioral alterations were accompanied by differential effects of stress on AEA levels in the amygdala and MAGL expression in the spinal cord between SD and WKY rats (Jennings et al., 2015). These data indicate that changes in the tone of the endocannabinoid system in the amygdala and spinal cord may underlie the differential effects of stress on inflammatory pain behavior between SD and WKY rats.

The chronic unpredictable stress (CUS) model of depression has been shown to exhibit thermal hyperalgesia in the hotplate test (Lomazzo et al., 2015), cold allodynia (Bravo et al., 2012), exacerbated trigeminovascular nociception (Zhang et al., 2013), inflammatory hyperalgesia in response to formalin administration (Shi et al., 2010), and persistent mechanical hyperalgesia following nerve growth factor administration (Lomazzo et al., 2015). Exposure to CUS has been shown to result in site-specific alterations in the endocannabinoid system, notably a downregulation of CB_1_ receptors, reduction in 2-AG levels and increased FAAH levels in the hippocampus (Hill et al., 2005; Reich et al., 2009), an increase in CB_1_ receptor mRNA expression in prefrontal cortex and decrease in expression in the midbrain (Bortolato et al., 2007), a decrease in CB_1_ receptor density in the hypothalamus and striatum and increased CB_1_ receptor density in the prefrontal cortex (Hill et al., 2008a; McLaughlin et al., 2013), a reduction in 2-AG–mediated retrograde synaptic transmission in the hippocampus (Zhong et al., 2014), and a reduction in AEA levels in the hypothalamus, prefrontal cortex, hippocampus, and striatum (Hill et al., 2008a). Depressive-like behaviors in the CUS model have been shown to be attenuated by endocannabinoid-modulating pharmacological agents, including the MAGL inhibitor JZL184 (Zhong et al., 2014; Zhang et al., 2015). Only one study to date has examined the effect of endocannabinoid modulation on affective and pain responding in CUS-exposed mice. Pretreatment with the FAAH inhibitor, URB597, or MAGL inhibitor, JZL184, which enhanced endogenous levels of AEA and 2-AG, respectively, significantly attenuated CUS-induced anxiety-related behavior in the light-dark box and concurrent thermal hyperalgesia (Lomazzo et al., 2015). Long-lasting widespread mechanical hyperalgesia, induced by intramuscular administration of nerve growth factor to CUS rats, was effectively reduced by URB597, but not JZL184 (Lomazzo et al., 2015). These data demonstrate an important role for AEA signaling in anxiety- and pain-related behavior in stress-exposed mice.

In addition to the evidence supporting a role for the endocannabinoid system in enhanced nociception observed in models of depression, alterations in endocannabinoid signaling have also been observed in animal models of chronic pain with comorbid alteration in affective responding (Table 3). A recent report by Racz and colleagues (2015) has revealed a prominent role of CB_1_-mediated events in affective behavior induced by neuropathic pain. In this study, partial sciatic nerve ligation (PNL) was employed to induce a model of neuropathic pain in wild-type and CB_1_^-/-^ mice. Wild-type and CB_1_^-/-^ mice exhibited mechanical allodynia following PNL. However, evaluation of anxiety- (light-dark test and the elevated zero-maze) and depressive-like (sucrose preference test) behavior 4 to 7 weeks following PNL revealed deficits in affective responding in CB_1_^-/-^, but not wild-type, mice (Racz et al., 2015). Thus, these data demonstrate that functionally active CB_1_ receptors confer resilience to pain-related anxiety/depression, highlighting a protective role for CB_1_ receptors against the emotional consequences of neuropathic pain. In a similar fashion, La Porta et al. (2015) recently investigated the role of the endocannabinoid system in affective and cognitive manifestations in an animal model of osteoarthritis. This study revealed that the anxiety-related behavior of osteoarthritic mice, identified in the elevated plus maze, was enhanced in CB_1_^-/-^ and absent in CB_2_^-/-^ mice, indicating differential effects of CB_1_ and CB_2_ receptors in mediating the affective dimension of pain in the model. Similar to effects in a neuropathic model (Racz et al., 2015), the data would indicate that CB_1_ receptors confer resilience, while CB_2_ receptors confer susceptibility to the development of arthritis-related anxiety. The authors suggest and provide some support that the differential effects of CB_1_ and CB_2_ receptors may be mediated by alterations in HPA axis functionality and responses (La Porta et al., 2015). In addition, this study also demonstrated that acute pharmacological blockade of CB_1_ or CB_2_ receptors ameliorated both the nociceptive and affective dimension of pain in the model (La Porta et al., 2015). Taken together, the data suggest that cortico-limbic endocannabinoid signaling is a key modulator of different osteoarthritis pain manifestations.

Only one study to date has investigated the role of CB_2_ receptors in the interaction between neuropathic pain and affective behavior. The chronic constriction injury model of neuropathic pain results in mechanical hypersensitivity and depressive-like behavior (immobility in the FST) in mice (Hu et al., 2009). Both depressive-like behavior and mechanical hyperalgesia following constriction injury were significantly attenuated by systemic administration of the CB_2_ receptor agonist GW405833, effects that were not observed in sham-operated animals. Furthermore, such behavioral effects were superior to administration of a tricyclic antidepressant, first line treatment for depression and chronic pain (Hu et al., 2009). The precise mechanism by which CB_2_ receptor agonism may elicit analgesic and antidepressant-like effects was not evaluated; however, given the well-recognized role for inflammatory processes in mediating chronic pain, it is possible that CB_2_ receptor activation attenuates such responses, preventing the development of central sensitization and mechanical allodynia and the associated increase in neuronal input to affective supraspinal sites. Further studies are required to evaluate this theory.

Intraperitoneal administration of a dilute concentration of lactic or acetic acid has been shown to induce abdominal stretching/writhing (visceral pain behavior), an effect associated with a reduction in feeding and hedonic behaviors (pain-depressed behavior). Evaluation of the role of the endocannabinoid system in mediating pain-stimulated and pain-depressed/suppressed responses in this model has revealed that genetic antagonism of CB_1_ receptors enhances acid-induced writhing (visceral pain stimulated behavior) and augments acid-induced reductions in feeding (Miller et al., 2011). In contrast, CB_1_ receptor agonism using Δ^9^-THC and CP55940 dose dependently inhibits acid-stimulated stretching while eliciting either no effect (Miller et al., 2012) or exacerbating (Kwilasz and Negus, 2012) acid-induced depression of feeding and scheduled controlled/intracranial self-stimulation in rats. Thus, under these conditions, potent synthetic cannabinoids such as Δ^9^-THC and CP55940 may elicit differential effects on visceral pain (attenuated) and pain-related depressive (exacerbated) behavior. However, the FAAH inhibitor URB597 exhibits a dose-related and CB_1_ receptor-mediated decrease in acid-stimulated stretching and suppression of feeding (Miller et al., 2012; Kwilasz et al., 2014). Furthermore, URB597 also elicits a delayed but significant attenuation of acid-induced suppression of intracranial self stimulation, an effect occurring independent of CB_1_ or CB_2_ mediation (Kwilasz et al., 2014). Taken together, these data indicate a role for CB_1_ receptors in mediating acid-induced visceral pain, with a possible common and/or alternative endocannabinoid mechanism mediating the associated anhedonic/depressive-like behavior.

Overall, despite the limited data, evidence suggests a prominent role for the endocannabinoid system in the interaction between depression and pain, although whether the effects are mediated by the same or parallel neuroanatomical pathways remains to be determined.

## Mechanisms By Which The Endocannabinoid System May Modulate Depression And Pain Interactions

While the exact mechanism(s) by which the endocannabinoid system may influence emotional and nociceptive processing remains undetermined, this system is known to elicit potent modulatory effects on neurotransmission, neuroendocrine, and inflammatory processes, all known to be altered in both depression and chronic pain. Presented here is an overview of how the endocannabinoid system may modulate affective and pain processing via interacting with these systems.

### Neurotransmitters

#### GABA and Glutamate

GABA- and glutamatergic neurotransmission are well recognized as important mediators in affect and nociceptive processing, and alterations in these systems have been demonstrated in both depression and chronic pain (for review, see Kendell et al., 2005; Rea et al., 2007). There are an increasing number of studies demonstrating that glutamatergic and GABAergic signaling play an important role in mediating the depressive symptoms associated with chronic pain. For example, ketamine, an NMDA receptor antagonist, attenuated depressive-like behavior following spared nerve injury without altering injury-induced hypersensitivity (Wang et al., 2011), while AMPAkines (which augment AMPA receptor function) have been shown to attenuate both pain hypersensitivity and associated depressive-like behavior in models of chronic inflammatory and neuropathic pain (Le et al., 2014). Furthermore, facilitation of glutamatergic transmission through AMPA receptors in the nucleus accumbens, a brain region involved in reward, resulted in attenuation of depression-like behavior in an animal model of neuropathic pain (Goffer et al., 2013). Despite a recognized role for the GABAergic system in emotional and pain processes, to our knowledge few studies have investigated the role of this system in affect-pain interactions to date. One such study has reported that GABA_A_ receptor activation in the RVM blocked formalin-induced hyperalgesia produced upon removal from an aversive elevated plus maze (stressor) (Cornelio et al., 2012). In addition, Quintero and colleagues (2011) have also identified that repeated forced swim stress-induced inflammatory hyperalgesia is initiated by decreased and delayed GABA release and GABA_A_ receptor activation and maintained by increased glutamate release and NMDA activation at the spinal cord level (Suarez-Roca et al., 2008).

CB_1_ receptors are expressed at a particularly high density on presynaptic nerve terminals of GABAergic and glutamatergic synapses in cortical and limbic areas of the brain associated with stress, emotional response, and pain modulation (Herkenham et al., 1990; Katona et al., 2001; Domenici et al., 2006; Wittmann et al., 2007). Endocannabinoids have been shown to exert behavioral effects via CB_1_ receptor agonism and resultant presynaptic inhibition of GABAergic and glutamatergic transmission (Meng et al., 1998; Millan, 2002) in supraspinal and spinal regions (Ulugol, 2014). In addition, glutamatergic neurotransmission is known to enhance endocannabinoid formation and subsequent CB_1_ receptor activation (Galante and Diana, 2004). Thus, complex bidirectional interaction exists between the endocannabinoid-glutamatergic-GABAergic systems. Several studies have demonstrated that CB_1_ modulation of GABAergic signaling is important in nociceptive (Manning et al., 2003; Naderi et al., 2005; Pernia-Andrade et al., 2009) and emotional (Haller et al., 2007; Naderi et al., 2008; Rossi et al., 2010; Rey et al., 2012; Reich et al., 2013) processing. Although it would appear intuitive, it is unknown if endocannabinoid modulation of GABA and/or glutamate plays a role in depression-pain interactions. However, we have demonstrated that CB_1_ receptors play an important role in mediating analgesia in response to acute stress (contextual fear conditioning) (Finn et al., 2004; Butler et al., 2008) and demonstrated an important role for GABAergic and glutamatergic signaling in the basolateral amygdala in mediating this effect (Rea et al., 2014). Thus, endocannabinoid modulation of GABAergic and glutamatergic tone can mediate stress-pain interactions and therefore may play a prominent role in coexistent psychiatric and pain disorders.

#### Monoamines

In addition to the treatment of depression, monoamine-based antidepressants are now regarded as first-line therapy for fibromyalgia and neuropathic pain. The monoaminergic system has been proposed as a common neural substrate for depression-pain associations. In accordance, a number of preclinical studies have demonstrated beneficial effects of modulating monoaminergic tone on depression associated with chronic pain and vice versa. For example, recent studies have demonstrated that chronic administration of 3-(4-fluorophenylselenyl)-2,5-diphenylselenophene, which increases serotonergic neurotransmission by inhibiting presynaptic serotonin transport, attenuates mechanical allodynia and depressive-like behavior in an animal model of neuropathic pain (Gai et al., 2014). Furthermore, chronic imipramine treatment reduces depressive-like behavior, but not hyperalgesia, in a rat model of neuropathic pain, effects mediated by increasing the neurotrophin BDNF (Yasuda et al., 2014). In addition, direct administration of a BDNF inducer (4-MC) into the brain attenuated thermal hyperalgesia and depressive-like behavior following nerve injury (Fukuhara et al., 2012; Ishikawa et al., 2014). The spinal serotonergic system has been shown to participate in the thermal hyperalgesia response in an animal model of depression, the olfactory bulbectomized rat (Rodriguez-Gaztelumendi et al., 2014). We have shown recently chronic amitriptyline treatment elicits an antidepressant-like effect and potently attenuates nerve injury-induced mechanical and cold allodynia in the bulbectomy model of depression (Burke et al., 2015). Thus, enhancing monoaminergic tone, and consequently central BDNF expression, modulates pain-depression behaviors.

Several lines of evidence have demonstrated that chronic antidepressant administration modulates endocannabinoid signaling (Hill et al., 2006, 2008b; Mato et al., 2010; Smaga et al., 2014), which underlie at least in part the mechanism by which these pharmacological agents modulate affective and nociceptive processes. Furthermore, endocannabinoid-induced modulation of serotonergic, noradrenergic, and dopaminergic transmission has been thoroughly investigated in several excellent reviews (Haj-Dahmane and Shen, 2011; Melis and Pistis, 2012; Kirilly et al., 2013). CB_1_ receptors are highly expressed on serotonergic, noradrenergic, and dopaminergic neurons and play an important role in the regulation of monoaminergic activity. Local and systemic administration of exogenous CB_1_ receptor agonists significantly increases serotonin (Bambico et al., 2007), noradrenaline (Jentsch et al., 1997; Oropeza et al., 2005; Page et al., 2007, 2008), and dopamine (Cheer et al., 2004; Solinas et al., 2006) levels in discrete brain regions that mediate emotional and nociceptive processing. Increasing endogenous levels of AEA and 2-AG through systemic administration of FAAH or MAGL inhibitors, respectively, has also been shown to enhance serotonergic and dopaminergic activity (Gobbi et al., 2005; Seif et al., 2011), and FAAH inhibition in the PFC increases serotonergic neuronal firing in the dorsal raphe nucleus (McLaughlin et al., 2012). In addition, endocannabinoids can inhibit the activity of monoamine oxidase (Fisar, 2010), the enzyme responsible for the metabolism of monoamines, which may also contribute to the increasing synaptic availability of the monoamines. Endocannabinoid activation of the CB_1_ receptor has been shown to control the function and expression of specific serotonin receptors, namely 5-HT_1A_, 5-HT_2A_, and 5-HT_2C_, in discrete regions of the CNS (Aso et al., 2009; Moranta et al., 2009; Zavitsanou et al., 2010; Franklin et al., 2013). Furthermore, spinal noradrenergic depletion is associated with compromised analgesic effects of CB_1_ agonism on formalin-evoked inflammatory pain (Gutierrez et al., 2003), while CB_1_ receptor activation results in attenuation of enhanced serotonergic firing in the dorsal raphe following nerve injury, an effects accompanied by antinociception (Palazzo et al., 2006). Taken together, these data demonstrate that cannabinoids modulate nociceptive tone, in part through modulation of noradrenergic and serotonergic systems. Thus, endocannabinoid-induced enhancement of monoaminergic tone may modulate emotional and nociceptive processes and thus the interaction between depression and pain.

#### Opioids

Numerous studies have characterized the causative role and therapeutic potential of opioidergic signaling in affective and nociceptive processing (for review, see Maletic and Raison, 2009; Lutz and Kieffer, 2013). Furthermore, alterations in opioid signaling in key brain regions such as the amygdala have been reported in a chronic pain model that exhibits comorbid anxiety-related behavior (Narita et al., 2006). Acute morphine administration attenuated both mechanical allodynia and anxiety-related behavior in the complete Freund’s adjuvant model of inflammatory pain (Parent et al., 2012). A very substantial body of evidence is now available to suggest that the endocannabinoid and opioidergic systems interact in therapeutically beneficial ways. CB_1_ and µ-opioid receptors are highly colocalized on neurons in areas of the brain associated with emotional and pain processing such as the caudate putamen, periaqueductal gray, and spinal cord (Rodriguez et al., 2001; Salio et al., 2001; Wilson-Poe et al., 2012). Coadministration of opioids and cannabinoids results in synergistic and bidirectional antinociceptive effects in several animal models (Cichewicz et al., 1999; Cichewicz and McCarthy, 2003; Tham et al., 2005; Roberts et al., 2006; Smith et al., 2007; Wilson et al., 2008; Wilson-Poe et al., 2013). In addition, increasing endocannabinoid tone has been shown to attenuate withdrawal symptoms in morphine-dependent animals (Smith et al., 2007; Wilson et al., 2008; Shahidi and Hasanein, 2011). Interestingly, cross tolerance also exists between these neuromodulatory systems. For example, decreases in the analgesic effects of Δ^9^-THC have been identified in morphine-tolerant animals and vice versa (Thorat and Bhargava, 1994). Furthermore, inhibition of opioid signalling (via ĸ-opioid receptors) attenuates the antidepressant-like effect of rimonabant (CB_1_ receptor antagonist/inverse agonist) in the FST (Lockie et al., 2011), and conversely the antidepressant-like effects of ĸ-opioid receptor antagonism are attenuated by the CB_1_ receptor antagonist/inverse agonist AM251 (Braida et al., 2009). Although further studies are required, collectively these findings suggest a regulatory role of the endocannabinoid system on opioid transmission, which may underlie the maintenance of coexistent depression-pain processes.

### Neuroendocrine Activity – HPA Axis

Dysregulation of the HPA axis has been implicated in the pathophysiology of both depression and pain disorders for decades (for review, see Bomholt et al., 2004; Vierck, 2006; Maric and Adzic, 2013; Belvederi Murri et al., 2014) and thus has also been proposed as a possible mediator in the depression-pain dyad (for review, see Blackburn-Munro, 2004). Several clinical studies have identified altered HPA axis activity in patients exhibiting symptoms of both depression and pain. For example, a cross-sectional study of patients with advanced breast cancer revealed increasing plasma cortisol levels that positively correlated with symptoms of depression and pain (Thornton et al., 2010). However in patients with fibromyalgia, enhanced cortisol release and dysregulation of HPA function associates with depressive, but not pain, symptoms, implying possible diverging mechanisms for both affective and nociceptive processing in this condition (Wingenfeld et al., 2010). Preclinical evidence of this reciprocity is also evident in an experimental model of gastritis, which is associated with gastrointestinal inflammation, pain, and anxiety- and depressive-like behaviors in rats (Luo et al., 2013). These behavioral alterations are accompanied by dysregulation of the HPA axis, characterized by increased expression of corticotrophin-releasing factor (CRF) mRNA and reduced expression of glucocorticoid receptor in the hypothalamus and increased plasma levels of corticosterone (Luo et al., 2013). Increased expression of CRF has also been reported in the paraventricular nucleus of the hypothalamus and dorsal raphe nucleus of WKY rats and animals preexposed to neonatal maternal separation, respectively (Bravo et al., 2011), two models of depression and associated hyperalgesia. Pharmacological blockade of CRF1 following intraamygdalar infusion of the CRF1 antagonist CP376395 inhibits hyperalgesic responding to colorectal distention in WKY rats, an effect not observed following glucocorticoid receptor or mineralocorticoid receptor antagonism (Johnson et al., 2012). Furthermore, the hyperalgesic visceromotor response to phasic colorectal distension following repeated water avoidance stress has been shown to be attenuated by CRF1 antagonism (Larauche et al., 2008). In addition, systemic or intraamygdalar injection of the CRF1 receptor antagonist NBI27914 blocks anxiety-related and nocifensive behavior in a rat model of arthritis (Ji et al., 2007). Thus, the CRF-HPA stress axis has been shown to play a key role in affective and/or nociceptive processing.

Several lines of evidence now support an important role for the endocannabinoid system as a modulator of HPA axis function and vice versa (for review, see Finn, 2010; Riebe and Wotjak, 2011; Hill and Tasker, 2012). The majority of evidence collated to date would suggest that basal HPA activity is under tonic inhibitory control by CB_1_ receptors. This has been shown in numerous reports where genetic deletion or pharmacological blockade of the CB_1_ receptor in vivo enhances expression of CRF and reduces glucocorticoid receptor expression in the hypothalamus and pituitary gland, respectively (Cota et al., 2007), and increases circulating levels of corticosterone and adrenocorticotropic hormone (Barna et al., 2004; Cota et al., 2007; Steiner et al., 2008). In addition, stress-induced increases in CRF expression in the paraventricular nucleus of the hypothalamus and the basolateral amygdala, as well as corticosterone secretion, are effectively blocked by pharmacological enhancement of endocannabinoid levels (Patel et al., 2004; Hill et al., 2009a; Bedse et al., 2014; Roberts et al., 2014), thus implying a role for endocannabinoid-CB_1_ receptor signaling in diminished hyperactivity of the HPA axis. Furthermore, recent evidence has shown that CRF1 activation in the amygdala induces FAAH and reduces AEA levels, an effect associated with anxiety-related behavior (Gray et al., 2015). Given the important role of the amygdala in affective modulation of pain, it is possible that CRF-mediated FAAH activation in this region may also modulate nociceptive possessing and associated emotional alterations. While there have been a few studies examining endocannabinoid-HPA axis effects in mediating the effects of the stress response (Hill et al., 2011; Roberts et al., 2014), to date no study has investigated if cannabinoid-mediated alterations of the HPA axis underlie alterations in nociceptive and/or affective behavior observed in depression-pain comorbidity.

### Neuro-Inflammatory Processes

Increasing evidence indicates a potent and prominent interaction between inflammation, depression, and pain (for review, see Walker et al., 2014). For instance, there is a high prevalence of depression among patients with inflammatory pain disorders such as fibromyalgia, arthritis, and irritable bowel disorder (Kappelman et al., 2014; Scheidt et al., 2014; Lin et al., 2015). In addition, patients receiving cytokine therapy for specific cancers and malignancies also develop depressive and/or painful symptomatology (Capuron and Ravaud, 1999; Capuron et al., 2001; Nogueira et al., 2012). Furthermore, increases in serum and cerebrospinal fluid levels of proinflammatory cytokines have been widely reported in both depression (Tuglu et al., 2003; Knuth et al., 2014; Bay-Richter et al., 2015) and pain conditions (Koch et al., 2007; Ludwig et al., 2008; Kadetoff et al., 2012). Inflammatory processes have also been shown to underlie the interaction between depression and pain in several animal models. For instance, increased expression of proinflammatory cytokines, concomitant with depressive-like behavior, has been identified in animal models of inflammatory (Kim et al., 2012; Maciel et al., 2013) and neuropathic (Norman et al., 2010; Burke et al., 2014; Dellarole et al., 2014; Zhou et al., 2015) pain. The innate inflammatory cascade has been shown to increase glutamate neurotransmission, central sensitization, and excitotoxicity, reduce BDNF and neurogenesis, and activate neurodegenerative cascades, events observed in both depression and pain conditions (for review, see Dantzer et al., 2011; Maes et al., 2011; Song and Wang, 2011; Zunszain et al., 2013; Walker et al., 2014).

Over the past decade, a wealth of data has demonstrated an important role for the endocannabinoid system in modulating innate immune function and inflammatory processes (for review, see Alhouayek and Muccioli, 2012; Zajkowska et al., 2014; Henry et al., 2015). Interactions between the endocannabinoid system and inflammatory mediators has been shown to influence synaptic transmission and neuronal function (Rossi et al., 2014). Spinal cord injury has been shown to be associated with increased coexpression of CB_1_ receptors with chemokines CCL2, CCL3, and/or CCR2 in the hippocampus, thalamus, and periaqueductal grey, areas associated with affective pain responding (Knerlich-Lukoschus et al., 2011), and studies have also demonstrated that CB_1_-chemokine interactions in the periaqueductal grey can modulate nociceptive responding (Benamar et al., 2008). Pharmacological enhancement of endocannabinoid tone also modulates inflammatory effects in vivo. For example, the FAAH inhibitor URB597 and the MAGL inhibitor JZL184 attenuate inflammation-induced astrocyte and microglial activation (Katz et al., 2015) and neuroinflammatory processes (Kerr et al., 2012, 2013; Henry et al., 2014). Furthermore, Zoppi and colleagues (2011, 2014) have demonstrated that pharmacological activation of CB_1_ or CB_2_ receptors attenuates, while genetic deletion of these receptors augments, repeated stress-induced proinflammatory responses in the frontal cortex. In addition, several studies have demonstrated that the analgesic effects of cannabinoids in chronic inflammatory and neuropathic pain are at least partially mediated by modulation of inflammatory responses (Burgos et al., 2012; Wilkerson et al., 2012; Burston et al., 2013; Lu et al., 2015). While there are no studies to date investigating if cannabinoid modulation of inflammatory processes underlies coexistent depressive and pain behavior, the above findings suggest a potential role for cannabinoid-mediated immunomodulation in the pathogenesis and treatment of co-occurring depression and pain.

## Conclusion and Future Directions

This review has provided an overview of the clinical and preclinical evidence supporting an association between depression and pain and vice versa. While a number of neural substrates have been proposed to underlie this association, this review provides a synthesis of the data supporting the contention that comorbid depression and pain may be mediated at least in part via dysregulation of the endocannabinoid system. Targeting the endocannabinoid system for therapeutic benefit has been an ever-expanding area of research with more than 150 clinical trials during the past decade evaluating the effects of cannabinoids in pain and psychiatric disorders (International Clinical Trials Registry Platform). While no study to date has specifically evaluated the effects of cannabinoids on depression-pain comorbidity, several have examined effects on mood and quality of life in patients receiving cannabinoid-based pharmaceuticals for analgesic purposes (Maida et al., 2008; Skrabek et al., 2008; Weber et al., 2009; Cameron et al., 2014), providing a basis for further study in this area. However, many of these cannabinoids are potent CB_1_ receptor agonists (Δ^9^-THC derivatives), an effect that may limit their usefulness in psychiatric conditions due to the associated adverse CNS effects. Cannabidiol has been shown to limit the adverse CNS effects associated with CB_1_ receptor agonists, and Sativex (1:1 Δ^9^-THC:cannabidiol) has been shown to reduce chronic pain and improve mood (Selvarajah et al., 2010; Vermersch, 2011). Thus, combination therapy may be a beneficial treatment strategy for depression-pain comorbidity. As highlighted throughout this review, modulation of endogenous cannabinoid tone provides an alternative to direct CB_1_ receptor agonism and although still in the early stages of clinical investigation, FAAH inhibitors such as PF-04457845 have demonstrated safety and tolerability in patients, although no effect on pain associated with osteoarthritis was reported (Huggins et al., 2012). However, this inhibitor is currently under clinical investigation for treatment of cannabis withdrawal, PTSD, and Tourette syndrome (International Clinical Trials Registry Platform), the results from which will provide important clinical data on the effect of FAAH inhibition on affective responding. Further clinical studies will provide greater insight into alterations and the role of the endocannabinoid system in the association between depression and pain.

Preclinical models that encapsulate the clinical scenario are particularly useful in gaining greater understanding of the neurobiology underlying depression-pain interactions. This review has presented the evidence to date demonstrating alterations in various components of the endocannabinoid system in models of depression-pain comorbidity and highlighted a particular role for AEA and CB_1_ receptors in mediating and modulating the affective and nociceptive processes in these models. However, research in this area is still in its infancy, and this review highlights the gaps in the knowledge and outstanding questions that remain to be addressed. For example, there have been limited data examining the role of other components of the endocannabinoid system on depression-pain interactions (2-AG, CB_2_, peroxisome proliferator-activated receptors, etc.), whether the endocannabinoid modulation of affect and nociception occur through the same or parallel pathways, and the mechanism by which the endocannabinoid system may mediate its effects (neurotransmitters, HPA axis, inflammation, or a combination). Furthermore, it is unknown if the role of the endocannabinoid system in chronic pain associated with depression is the same or different from altered affective processing associated with chronic pain conditions. Such studies are essential if we are to move towards a more comprehensive understanding of the neurobiology underlying the association between these pain and depression and fully explore the potential clinical efficacy of targeting the endocannabinoid system for resolution of these comorbid conditions.

## Statement of Interest

None.

## References

[CIT0001] Agrawal A Nelson EC Littlefield AK Bucholz KK Degenhardt L Henders AK Madden PA Martin NG Montgomery GW Pergadia ML Sher KJ Heath AC Lynskey MT (2012) Cannabinoid receptor genotype moderation of the effects of childhood physical abuse on anhedonia and depression. Arch Gen Psychiatry 69:732–740. 22393204 10.1001/archgenpsychiatry.2011.2273 PMC3706194

[CIT0002] Alhouayek M Muccioli GG (2012) The endocannabinoid system in inflammatory bowel diseases: from pathophysiology to therapeutic opportunity. Trends Mol Med 18:615–625. 22917662 10.1016/j.molmed.2012.07.009

[CIT0003] Anderson G Maes M Berk M (2012) Inflammation-related disorders in the tryptophan catabolite pathway in depression and somatization. Adv Protein Chem Struct Biol 88:27–48. 22814705 10.1016/B978-0-12-398314-5.00002-7

[CIT0004] Arora V Kuhad A Tiwari V Chopra K (2011) Curcumin ameliorates reserpine-induced pain-depression dyad: behavioral, biochemical, neurochemical and molecular evidences. Psychoneuroendocrinology 36:1570–1581. 21612876 10.1016/j.psyneuen.2011.04.012

[CIT0005] Ashton CH Moore PB (2011) Endocannabinoid system dysfunction in mood and related disorders. Acta Psychiatr Scand 124:250–261. 21916860 10.1111/j.1600-0447.2011.01687.x

[CIT0006] Aso E Renoir T Mengod G Ledent C Hamon M Maldonado R Lanfumey L Valverde O (2009) Lack of CB1 receptor activity impairs serotonergic negative feedback. J Neurochem 109:935–944. 19302195 10.1111/j.1471-4159.2009.06025.x

[CIT0007] Atwood BK Straiker A Mackie K (2012) CB(2) cannabinoid receptors inhibit synaptic transmission when expressed in cultured autaptic neurons. Neuropharmacology 63:514–523. 22579668 10.1016/j.neuropharm.2012.04.024 PMC3392362

[CIT0008] Baek JH Zheng Y Darlington CL Smith PF (2008) Cannabinoid CB2 receptor expression in the rat brainstem cochlear and vestibular nuclei. Acta Otolaryngol 128:961–967. 19086305 10.1080/00016480701796944

[CIT0009] Bambico FR Katz N Debonnel G Gobbi G (2007) Cannabinoids elicit antidepressant-like behavior and activate serotonergic neurons through the medial prefrontal cortex. J Neurosci 27:11700–11711. 17959812 10.1523/JNEUROSCI.1636-07.2007 PMC6673235

[CIT0010] Bar KJ Brehm S Boettger MK Boettger S Wagner G Sauer H (2005) Pain perception in major depression depends on pain modality. Pain 117:97–103. 16061323 10.1016/j.pain.2005.05.016

[CIT0011] Bardin L Malfetes N Newman-Tancredi A Depoortere R (2009) Chronic restraint stress induces mechanical and cold allodynia, and enhances inflammatory pain in rat: relevance to human stress-associated painful pathologies. Behav Brain Res 205:360–366. 19616033 10.1016/j.bbr.2009.07.005

[CIT0012] Barna I Zelena D Arszovszki AC Ledent C (2004) The role of endogenous cannabinoids in the hypothalamo-pituitary-adrenal axis regulation: in vivo and in vitro studies in CB1 receptor knockout mice. Life Sci 75:2959–2970. 15454346 10.1016/j.lfs.2004.06.006

[CIT0013] Bay-Richter C Linderholm KR Lim CK Samuelsson M Traskman-Bendz L Guillemin GJ Erhardt S Brundin L (2015) A role for inflammatory metabolites as modulators of the glutamate N-methyl-D-aspartate receptor in depression and suicidality. Brain Behav Immun 43:110–117. 25124710 10.1016/j.bbi.2014.07.012

[CIT0014] Bedse G Colangeli R Lavecchia AM Romano A Altieri F Cifani C Cassano T Gaetani S (2014) Role of the basolateral amygdala in mediating the effects of the fatty acid amide hydrolase inhibitor URB597 on HPA axis response to stress. Eur Neuropsychopharmacol 24:1511–1523. 25106694 10.1016/j.euroneuro.2014.07.005

[CIT0015] Belvederi Murri M Pariante C Mondelli V Masotti M Atti AR Mellacqua Z Antonioli M Ghio L Menchetti M Zanetidou S Innamorati M Amore M (2014) HPA axis and aging in depression: systematic review and meta-analysis. Psychoneuroendocrinology 41:46–62. 24495607 10.1016/j.psyneuen.2013.12.004

[CIT0016] Benamar K Geller EB Adler MW (2008) First in vivo evidence for a functional interaction between chemokine and cannabinoid systems in the brain. J Pharmacol Exp Ther 325:641–645. 18281594 10.1124/jpet.107.135053

[CIT0017] Ben-Tovim DI Schwartz MS (1981) Hypoalgesia in depressive illness. Br J Psychiatry 138:37–39. 7272643 10.1192/bjp.138.1.37

[CIT0018] Berdyshev EV (2000) Cannabinoid receptors and the regulation of immune response. Chem Phys Lipids 108:169–190. 11106790 10.1016/s0009-3084(00)00195-x

[CIT0019] Blackburn-Munro G (2004) Hypothalamo-pituitary-adrenal axis dysfunction as a contributory factor to chronic pain and depression. Curr Pain Headache Rep 8:116–124. 14980146 10.1007/s11916-004-0025-9

[CIT0020] Blankman JL Simon GM Cravatt BF (2007) A comprehensive profile of brain enzymes that hydrolyze the endocannabinoid 2-arachidonoylglycerol. Chem Biol 14:1347–1356. 18096503 10.1016/j.chembiol.2007.11.006 PMC2692834

[CIT0021] Boettger MK Grossmann D Bar KJ (2013) Thresholds and perception of cold pain, heat pain, and the thermal grill illusion in patients with major depressive disorder. Psychosom Med 75:281–287. 23460720 10.1097/PSY.0b013e3182881a9c

[CIT0022] Bomholt SF Harbuz MS Blackburn-Munro G Blackburn-Munro RE (2004) Involvement and role of the hypothalamo-pituitary-adrenal (HPA) stress axis in animal models of chronic pain and inflammation. Stress 7:1–14. 15204028 10.1080/10253890310001650268

[CIT0023] Borges G Neto F Mico JA Berrocoso E (2014) Reversal of monoarthritis-induced affective disorders by diclofenac in rats. Anesthesiology 120:1476–1490. 24534905 10.1097/ALN.0000000000000177

[CIT0024] Bortolato M Mangieri RA Fu J Kim JH Arguello O Duranti A Tontini A Mor M Tarzia G Piomelli D (2007) Antidepressant-like activity of the fatty acid amide hydrolase inhibitor URB597 in a rat model of chronic mild stress. Biol Psychiatry 62:1103–1110. 17511970 10.1016/j.biopsych.2006.12.001

[CIT0025] Boychuk DG Goddard G Mauro G Orellana MF (2015) The effectiveness of cannabinoids in the management of chronic nonmalignant neuropathic pain: a systematic review. J Oral Facial Pain Headache 29:7–14. 25635955 10.11607/ofph.1274

[CIT0026] Braida D Capurro V Zani A Rubino T Vigano D Parolaro D Sala M (2009) Potential anxiolytic- and antidepressant-like effects of salvinorin A, the main active ingredient of Salvia divinorum, in rodents. Br J Pharmacol 157:844–853. 19422370 10.1111/j.1476-5381.2009.00230.x PMC2721268

[CIT0027] Bravo JA Dinan TG Cryan JF (2011) Alterations in the central CRF system of two different rat models of comorbid depression and functional gastrointestinal disorders. Int J Neuropsychopharmacol 14:666–683. 20860876 10.1017/S1461145710000994

[CIT0028] Bravo L Mico JA Rey-Brea R Perez-Nievas B Leza JC Berrocoso E (2012) Depressive-like states heighten the aversion to painful stimuli in a rat model of comorbid chronic pain and depression. Anesthesiology 117:613–625. 22846678 10.1097/ALN.0b013e3182657b3e

[CIT0029] Bravo L Torres-Sanchez S Alba-Delgado C Mico JA Berrocoso E (2014) Pain exacerbates chronic mild stress-induced changes in noradrenergic transmission in rats. Eur Neuropsychopharmacol 24:996–1003. 24491949 10.1016/j.euroneuro.2014.01.011

[CIT0030] Burgos E Gomez-Nicola D Pascual D Martin MI Nieto-Sampedro M Goicoechea C (2012) Cannabinoid agonist WIN 55,212-2 prevents the development of paclitaxel-induced peripheral neuropathy in rats. Possible involvement of spinal glial cells. Eur J Pharmacol 682:62–72. 22374260 10.1016/j.ejphar.2012.02.008

[CIT0031] Burke NN Hayes E Calpin P Kerr DM Moriarty O Finn DP Roche M (2010) Enhanced nociceptive responding in two rat models of depression is associated with alterations in monoamine levels in discrete brain regions. Neuroscience 171:1300–1313. 20955767 10.1016/j.neuroscience.2010.10.030

[CIT0032] Burke NN Geoghegan E Kerr DM Moriarty O Finn DP Roche M (2013) Altered neuropathic pain behavior in a rat model of depression is associated with changes in inflammatory gene expression in the amygdala. Genes Brain Behav 12:705–713. 23957449 10.1111/gbb.12080

[CIT0033] Burke NN Kerr DM Moriarty O Finn DP Roche M (2014) Minocycline modulates neuropathic pain behavior and cortical M1-M2 microglial gene expression in a rat model of depression. Brain Behav Immun 42:147–156. 24994592 10.1016/j.bbi.2014.06.015

[CIT0034] Burke NN Finn DP Roche M (2015) Chronic administration of amitriptyline differentially alters neuropathic pain-related behavior in the presence and absence of a depressive-like phenotype. Behav Brain Res 278:193–201. 25300472 10.1016/j.bbr.2014.09.044

[CIT0035] Burston JJ Sagar DR Shao P Bai M King E Brailsford L Turner JM Hathway GJ Bennett AJ Walsh DA Kendall DA Lichtman A Chapman V (2013) Cannabinoid CB2 receptors regulate central sensitization and pain responses associated with osteoarthritis of the knee joint. PLoS One 8:e80440. 24282543 10.1371/journal.pone.0080440 PMC3840025

[CIT0036] Butler RK Rea K Lang Y Gavin AM Finn DP (2008) Endocannabinoid-mediated enhancement of fear-conditioned analgesia in rats: opioid receptor dependency and molecular correlates. Pain 140:491–500. 19004548 10.1016/j.pain.2008.10.002

[CIT0037] Cameron C Watson D Robinson J (2014) Use of a synthetic cannabinoid in a correctional population for posttraumatic stress disorder-related insomnia and nightmares, chronic pain, harm reduction, and other indications: a retrospective evaluation. J Clin Psychopharmacol 34:559–564. 24987795 10.1097/JCP.0000000000000180 PMC4165471

[CIT0038] Capuron L Ravaud A (1999) Prediction of the depressive effects of interferon alfa therapy by the patient’s initial affective state. N Engl J Med 340:1370. 10223879 10.1056/NEJM199904293401716

[CIT0039] Capuron L Ravaud A Gualde N Bosmans E Dantzer R Maes M Neveu PJ (2001) Association between immune activation and early depressive symptoms in cancer patients treated with interleukin-2-based therapy. Psychoneuroendocrinology 26:797–808. 11585680 10.1016/s0306-4530(01)00030-0

[CIT0040] Carlisle SJ Marciano-Cabral F Staab A Ludwick C Cabral GA (2002) Differential expression of the CB2 cannabinoid receptor by rodent macrophages and macrophage-like cells in relation to cell activation. Int Immunopharmacol 2:69–82. 11789671 10.1016/s1567-5769(01)00147-3

[CIT0041] Cavuoto P McAinch AJ Hatzinikolas G Janovska A Game P Wittert GA (2007) The expression of receptors for endocannabinoids in human and rodent skeletal muscle. Biochem Biophys Res Commun 364:105–110. 17935697 10.1016/j.bbrc.2007.09.099

[CIT0042] Cheer JF Wassum KM Heien ML Phillips PE Wightman RM (2004) Cannabinoids enhance subsecond dopamine release in the nucleus accumbens of awake rats. J Neurosci 24:4393–4400. 15128853 10.1523/JNEUROSCI.0529-04.2004 PMC6729440

[CIT0043] Cichewicz DL Martin ZL Smith FL Welch SP (1999) Enhancement mu opioid antinociception by oral delta9-tetrahydrocannabinol: dose-response analysis and receptor identification. J Pharmacol Exp Ther 289:859–867. 10215664

[CIT0044] Cichewicz DL McCarthy EA (2003) Antinociceptive synergy between delta(9)-tetrahydrocannabinol and opioids after oral administration. J Pharmacol Exp Ther 304:1010–1015. 12604676 10.1124/jpet.102.045575

[CIT0045] Cornelio AM Nunes-de-Souza RL Morgan MM (2012) Contribution of the rostral ventromedial medulla to post-anxiety induced hyperalgesia. Brain Res 1450:80–86. 22418057 10.1016/j.brainres.2012.02.037

[CIT0046] Cota D (2007) CB1 receptors: emerging evidence for central and peripheral mechanisms that regulate energy balance, metabolism, and cardiovascular health. Diabetes Metab Res Rev 23:507–517. 17683024 10.1002/dmrr.764

[CIT0047] Cota D Steiner MA Marsicano G Cervino C Herman JP Grubler Y Stalla J Pasquali R Lutz B Stalla GK Pagotto U (2007) Requirement of cannabinoid receptor type 1 for the basal modulation of hypothalamic-pituitary-adrenal axis function. Endocrinology 148:1574–1581. 17194743 10.1210/en.2005-1649

[CIT0048] Cravatt BF Giang DK Mayfield SP Boger DL Lerner RA Gilula NB (1996) Molecular characterization of an enzyme that degrades neuromodulatory fatty-acid amides. Nature 384:83–87. 8900284 10.1038/384083a0

[CIT0049] Dantzer R O’Connor JC Lawson MA Kelley KW (2011) Inflammation-associated depression: from serotonin to kynurenine. Psychoneuroendocrinology 36:426–436. 21041030 10.1016/j.psyneuen.2010.09.012 PMC3053088

[CIT0050] del Rey A Yau HJ Randolf A Centeno MV Wildmann J Martina M Besedovsky HO Apkarian AV (2011) Chronic neuropathic pain-like behavior correlates with IL-1beta expression and disrupts cytokine interactions in the hippocampus. Pain 152:2827–2835. 22033365 10.1016/j.pain.2011.09.013 PMC3215892

[CIT0051] Dellarole A Morton P Brambilla R Walters W Summers S Bernardes D Grilli M Bethea JR (2014) Neuropathic pain-induced depressive-like behavior and hippocampal neurogenesis and plasticity are dependent on TNFR1 signaling. Brain Behav Immun 41:65–81. 24938671 10.1016/j.bbi.2014.04.003 PMC4167189

[CIT0052] Demuth DG Molleman A (2006) Cannabinoid signalling. Life Sci 78:549–563. 16109430 10.1016/j.lfs.2005.05.055

[CIT0053] Devane WA Dysarz FA 3rd Johnson MR Melvin LS Howlett AC (1988) Determination and characterization of a cannabinoid receptor in rat brain. Mol Pharmacol 34:605–613. 2848184

[CIT0054] Devane WA Hanus L Breuer A Pertwee RG Stevenson LA Griffin G Gibson D Mandelbaum A Etinger A Mechoulam R (1992) Isolation and structure of a brain constituent that binds to the cannabinoid receptor. Science 258:1946–1949. 1470919 10.1126/science.1470919

[CIT0055] Di Marzo V Fontana A Cadas H Schinelli S Cimino G Schwartz JC Piomelli D (1994) Formation and inactivation of endogenous cannabinoid anandamide in central neurons. Nature 372:686–691. 7990962 10.1038/372686a0

[CIT0056] Doan L Manders T Wang J (2015) Neuroplasticity underlying the comorbidity of pain and depression. Neural Plast 2015:504691. 25810926 10.1155/2015/504691 PMC4355564

[CIT0057] Domenici MR Azad SC Marsicano G Schierloh A Wotjak CT Dodt HU Zieglgansberger W Lutz B Rammes G (2006) Cannabinoid receptor type 1 located on presynaptic terminals of principal neurons in the forebrain controls glutamatergic synaptic transmission. J Neurosci 26:5794–5799. 16723537 10.1523/JNEUROSCI.0372-06.2006 PMC6675276

[CIT0058] Domschke K Dannlowski U Ohrmann P Lawford B Bauer J Kugel H Heindel W Young R Morris P Arolt V Deckert J Suslow T Baune BT (2008) Cannabinoid receptor 1 (CNR1) gene: impact on antidepressant treatment response and emotion processing in major depression. Eur Neuropsychopharmacol 18:751–759. 18579347 10.1016/j.euroneuro.2008.05.003

[CIT0059] Emery PC Wilson KG Kowal J (2014) Major depressive disorder and sleep disturbance in patients with chronic pain. Pain Res Manag 19:35–41. 24367795 10.1155/2014/480859 PMC3938341

[CIT0060] Emptage NP Sturm R Robinson RL (2005) Depression and comorbid pain as predictors of disability, employment, insurance status, and health care costs. Psychiatr Serv 56:468–474. 15812099 10.1176/appi.ps.56.4.468

[CIT0061] Fichna J Wood JT Papanastasiou M Vadivel SK Oprocha P Salaga M Sobczak M Mokrowiecka A Cygankiewicz AI Zakrzewski PK Malecka-Panas E Krajewska WM Koscielniak P Makriyannis A Storr MA (2013) Endocannabinoid and cannabinoid-like fatty acid amide levels correlate with pain-related symptoms in patients with IBS-D and IBS-C: a pilot study. PLoS One 8:e85073. 24386448 10.1371/journal.pone.0085073 PMC3874007

[CIT0062] Finn DP Beckett SR Richardson D Kendall DA Marsden CA Chapman V (2004) Evidence for differential modulation of conditioned aversion and fear-conditioned analgesia by CB1 receptors. Eur J Neurosci 20:848–852. 15255996 10.1111/j.1460-9568.2004.03509.x

[CIT0063] Finn DP (2010) Endocannabinoid-mediated modulation of stress responses: physiological and pathophysiological significance. Immunobiology 215:629–646. 19616342 10.1016/j.imbio.2009.05.011

[CIT0064] Fisar Z (2010) Inhibition of monoamine oxidase activity by cannabinoids. Naunyn Schmiedebergs Arch Pharmacol 381:563–572. 20401651 10.1007/s00210-010-0517-6

[CIT0065] Franklin JM Mathew M Carrasco GA (2013) Cannabinoid-induced upregulation of serotonin 2A receptors in the hypothalamic paraventricular nucleus and anxiety-like behaviors in rats. Neurosci Lett 548:165–169. 23721787 10.1016/j.neulet.2013.05.039 PMC3735609

[CIT0066] Fukuhara K Ishikawa K Yasuda S Kishishita Y Kim HK Kakeda T Yamamoto M Norii T Ishikawa T (2012) Intracerebroventricular 4-methylcatechol (4-MC) ameliorates chronic pain associated with depression-like behavior via induction of brain-derived neurotrophic factor (BDNF). Cell Mol Neurobiol 32:971–977. 22198556 10.1007/s10571-011-9782-2 PMC11498583

[CIT0067] Gai BM Bortolatto CF Bruning CA Zborowski VA Stein AL Zeni G Nogueira CW (2014) Depression-related behavior and mechanical allodynia are blocked by 3-(4-fluorophenylselenyl)-2,5-diphenylselenophene in a mouse model of neuropathic pain induced by partial sciatic nerve ligation. Neuropharmacology 79:580–589. 24467850 10.1016/j.neuropharm.2014.01.020

[CIT0068] Galante M Diana MA (2004) Group I metabotropic glutamate receptors inhibit GABA release at interneuron-Purkinje cell synapses through endocannabinoid production. J Neurosci 24:4865–4874. 15152047 10.1523/JNEUROSCI.0403-04.2004 PMC6729473

[CIT0069] Galiegue S Mary S Marchand J Dussossoy D Carriere D Carayon P Bouaboula M Shire D Le Fur G Casellas P (1995) Expression of central and peripheral cannabinoid receptors in human immune tissues and leukocyte subpopulations. Eur J Biochem 232:54–61. 7556170 10.1111/j.1432-1033.1995.tb20780.x

[CIT0070] Gameiro GH Andrade Ada S de Castro M Pereira LF Tambeli CH Veiga MC (2005) The effects of restraint stress on nociceptive responses induced by formalin injected in rat’s TMJ. Pharmacol Biochem Behav 82:338–344. 16213578 10.1016/j.pbb.2005.09.003

[CIT0071] Gameroff MJ Olfson M (2006) Major depressive disorder, somatic pain, and health care costs in an urban primary care practice. J Clin Psychiatry 67:1232–1239. 16965201 10.4088/jcp.v67n0809

[CIT0072] Gaoni Y Mechoulam R (1964) Isolation, structure, and partial synthesis of an active constituent of hashish. J Am Chem Soc 86:1646.

[CIT0073] Gibney SM Gosselin RD Dinan TG Cryan JF (2010) Colorectal distension–induced prefrontal cortex activation in the Wistar-Kyoto rat: implications for irritable bowel syndrome. Neuroscience 165:675–683. 19765638 10.1016/j.neuroscience.2009.08.076

[CIT0074] Gobbi G Bambico FR Mangieri R Bortolato M Campolongo P Solinas M Cassano T Morgese MG Debonnel G Duranti A Tontini A Tarzia G Mor M Trezza V Goldberg SR Cuomo V Piomelli D (2005) Antidepressant-like activity and modulation of brain monoaminergic transmission by blockade of anandamide hydrolysis. Proc Natl Acad Sci U S A 102:18620–18625. 16352709 10.1073/pnas.0509591102 PMC1317988

[CIT0075] Goesling J Clauw DJ Hassett AL (2013) Pain and depression: an integrative review of neurobiological and psychological factors. Curr Psychiatry Rep 15:421. 24214740 10.1007/s11920-013-0421-0

[CIT0076] Goffer Y Xu D Eberle SE D’Amour J Lee M Tukey D Froemke RC Ziff EB Wang J (2013) Calcium-permeable AMPA receptors in the nucleus accumbens regulate depression-like behaviors in the chronic neuropathic pain state. J Neurosci 33:19034–19044. 24285907 10.1523/JNEUROSCI.2454-13.2013 PMC3841460

[CIT0077] Goncalves L Silva R Pinto-Ribeiro F Pego JM Bessa JM Pertovaara A Sousa N Almeida A (2008) Neuropathic pain is associated with depressive behavior and induces neuroplasticity in the amygdala of the rat. Exp Neurol 213:48–56. 18599044 10.1016/j.expneurol.2008.04.043

[CIT0078] Gong JP Onaivi ES Ishiguro H Liu QR Tagliaferro PA Brusco A Uhl GR (2006) Cannabinoid CB2 receptors: immunohistochemical localization in rat brain. Brain Res 1071:10–23. 16472786 10.1016/j.brainres.2005.11.035

[CIT0079] Gormsen L Ribe AR Raun P Rosenberg R Videbech P Vestergaard P Bach FW Jensen TS (2004) Pain thresholds during and after treatment of severe depression with electroconvulsive therapy. Eur J Pain 8:487–493. 15324780 10.1016/j.ejpain.2003.11.015

[CIT0080] Gorzalka BB Hill MN (2011) Putative role of endocannabinoid signaling in the etiology of depression and actions of antidepressants. Prog Neuropsychopharmacol Biol Psychiatry 35:1575–1585. 21111017 10.1016/j.pnpbp.2010.11.021

[CIT0081] Gosselin RD O’Connor RM Tramullas M Julio-Pieper M Dinan TG Cryan JF (2010) Riluzole normalizes early-life stress-induced visceral hypersensitivity in rats: role of spinal glutamate reuptake mechanisms. Gastroenterology 138:2418–2425. 20226190 10.1053/j.gastro.2010.03.003

[CIT0082] Gray JM Vecchiarelli HA Morena M Lee TT Hermanson DJ Kim AB McLaughlin RJ Hassan KI Kuhne C Wotjak CT Deussing JM Patel S Hill MN (2015) Corticotropin-releasing hormone drives anandamide hydrolysis in the amygdala to promote anxiety. J Neurosci 35:3879–3892. 25740517 10.1523/JNEUROSCI.2737-14.2015 PMC4348185

[CIT0083] Greenbaum L Tegeder I Barhum Y Melamed E Roditi Y Djaldetti R (2012) Contribution of genetic variants to pain susceptibility in Parkinson disease. Eur J Pain 16:1243–1250. 22473870 10.1002/j.1532-2149.2012.00134.x

[CIT0084] Grover S Kumar V Chakrabarti S Hollikatti P Singh P Tyagi S Kulhara P Avasthi A (2012) Prevalence and type of functional somatic complaints in patients with first-episode depression. East Asian Arch Psychiatry 22:146–153. 23271583

[CIT0085] Gutierrez T Nackley AG Neely MH Freeman KG Edwards GL Hohmann AG (2003) Effects of neurotoxic destruction of descending noradrenergic pathways on cannabinoid antinociception in models of acute and tonic nociception. Brain Res 987:176–185. 14499961 10.1016/s0006-8993(03)03324-9

[CIT0086] Haj-Dahmane S Shen RY (2011) Modulation of the serotonin system by endocannabinoid signaling. Neuropharmacology 61:414–420. 21354188 10.1016/j.neuropharm.2011.02.016 PMC3110547

[CIT0087] Haller J Matyas F Soproni K Varga B Barsy B Nemeth B Mikics E Freund TF Hajos N (2007) Correlated species differences in the effects of cannabinoid ligands on anxiety and on GABAergic and glutamatergic synaptic transmission. Eur J Neurosci 25:2445–2456. 17445240 10.1111/j.1460-9568.2007.05476.x PMC1890583

[CIT0088] Hanus L Abu-Lafi S Fride E Breuer A Vogel Z Shalev DE Kustanovich I Mechoulam R (2001) 2-arachidonyl glyceryl ether, an endogenous agonist of the cannabinoid CB1 receptor. Proc Natl Acad Sci U S A 98:3662–3665. 11259648 10.1073/pnas.061029898 PMC31108

[CIT0089] Hasnie FS Wallace VC Hefner K Holmes A Rice AS (2007) Mechanical and cold hypersensitivity in nerve-injured C57BL/6J mice is not associated with fear-avoidance- and depression-related behavior. Br J Anaesth 98:816–822. 17478455 10.1093/bja/aem087 PMC2656645

[CIT0090] Hassett AL Aquino JK Ilgen MA (2014) The risk of suicide mortality in chronic pain patients. Curr Pain Headache Rep 18:436. 24952608 10.1007/s11916-014-0436-1

[CIT0091] Hawker GA Gignac MA Badley E Davis AM French MR Li Y Perruccio AV Power JD Sale J Lou W (2011) A longitudinal study to explain the pain-depression link in older adults with osteoarthritis. Arthritis Care Res (Hoboken) 63:1382–1390. 20662042 10.1002/acr.20298

[CIT0092] Henry RJ Kerr DM Finn DP Roche M (2014) FAAH-mediated modulation of TLR3-induced neuroinflammation in the rat hippocampus. J Neuroimmunol 276:126–134. 25245162 10.1016/j.jneuroim.2014.09.002

[CIT0093] Henry RJ Kerr DM Finn DP Roche M (2015) For whom the endocannabinoid tolls: modulation of innate immune function and implications for psychiatric disorders. Prog Neuropsychopharmacol Biol Psychiatry PMID: 25794989. 10.1016/j.pnpbp.2015.03.006 25794989

[CIT0094] Herkenham M (1991) Characterization and localization of cannabinoid receptors in brain: an in vitro technique using slide-mounted tissue sections. NIDA Res Monogr 112:129–145. 1753996

[CIT0095] Herkenham M Lynn AB Little MD Johnson MR Melvin LS de Costa BR Rice KC (1990) Cannabinoid receptor localization in brain. Proc Natl Acad Sci U S A 87:1932–1936. 2308954 10.1073/pnas.87.5.1932 PMC53598

[CIT0096] Hill MN Gorzalka BB (2005) Is there a role for the endocannabinoid system in the etiology and treatment of melancholic depression? Behav Pharmacol 16:333–352. 16148438 10.1097/00008877-200509000-00006

[CIT0097] Hill MN Tasker JG (2012) Endocannabinoid signaling, glucocorticoid-mediated negative feedback, and regulation of the hypothalamic-pituitary-adrenal axis. Neuroscience 204:5–16. 22214537 10.1016/j.neuroscience.2011.12.030 PMC3288468

[CIT0098] Hill MN Patel S Carrier EJ Rademacher DJ Ormerod BK Hillard CJ Gorzalka BB (2005) Downregulation of endocannabinoid signaling in the hippocampus following chronic unpredictable stress. Neuropsychopharmacology 30:508–515. 15525997 10.1038/sj.npp.1300601

[CIT0099] Hill MN Ho WS Sinopoli KJ Viau V Hillard CJ Gorzalka BB (2006) Involvement of the endocannabinoid system in the ability of long-term tricyclic antidepressant treatment to suppress stress-induced activation of the hypothalamic-pituitary-adrenal axis. Neuropsychopharmacology 31:2591–2599. 16710317 10.1038/sj.npp.1301092

[CIT0100] Hill MN Carrier EJ McLaughlin RJ Morrish AC Meier SE Hillard CJ Gorzalka BB (2008a) Regional alterations in the endocannabinoid system in an animal model of depression: effects of concurrent antidepressant treatment. J Neurochem 106:2322–2336. 18643796 10.1111/j.1471-4159.2008.05567.x PMC2606621

[CIT0101] Hill MN Ho WS Hillard CJ Gorzalka BB (2008b) Differential effects of the antidepressants tranylcypromine and fluoxetine on limbic cannabinoid receptor binding and endocannabinoid contents. J Neural Transm 115:1673–1679. 18974922 10.1007/s00702-008-0131-7 PMC2992975

[CIT0102] Hill MN Miller GE Ho WS Gorzalka BB Hillard CJ (2008c) Serum endocannabinoid content is altered in females with depressive disorders: a preliminary report. Pharmacopsychiatry 41:48–53. 18311684 10.1055/s-2007-993211 PMC3422568

[CIT0103] Hill MN McLaughlin RJ Morrish AC Viau V Floresco SB Hillard CJ Gorzalka BB (2009a) Suppression of amygdalar endocannabinoid signaling by stress contributes to activation of the hypothalamic-pituitary-adrenal axis. Neuropsychopharmacology 34:2733–2745. 19710634 10.1038/npp.2009.114 PMC3197779

[CIT0104] Hill MN Miller GE Carrier EJ Gorzalka BB Hillard CJ (2009b) Circulating endocannabinoids and N-acyl ethanolamines are differentially regulated in major depression and following exposure to social stress. Psychoneuroendocrinology 34:1257–1262. 19394765 10.1016/j.psyneuen.2009.03.013 PMC2716432

[CIT0105] Hill MN McLaughlin RJ Pan B Fitzgerald ML Roberts CJ Lee TT Karatsoreos IN Mackie K Viau V Pickel VM McEwen BS Liu QS Gorzalka BB Hillard CJ (2011) Recruitment of prefrontal cortical endocannabinoid signaling by glucocorticoids contributes to termination of the stress response. J Neurosci 31:10506–10515. 21775596 10.1523/JNEUROSCI.0496-11.2011 PMC3179266

[CIT0106] Hillard CJ Liu QS (2014) Endocannabinoid signaling in the etiology and treatment of major depressive illness. Curr Pharm Des 20:3795–3811. 24180398 10.2174/13816128113196660735 PMC4002665

[CIT0107] Hu B Doods H Treede RD Ceci A (2009) Depression-like behavior in rats with mononeuropathy is reduced by the CB2-selective agonist GW405833. Pain 143:206–212. 19345493 10.1016/j.pain.2009.02.018

[CIT0108] Huang SM Bisogno T Trevisani M Al-Hayani A De Petrocellis L Fezza F Tognetto M Petros TJ Krey JF Chu CJ Miller JD Davies SN Geppetti P Walker JM Di Marzo V (2002) An endogenous capsaicin-like substance with high potency at recombinant and native vanilloid VR1 receptors. Proc Natl Acad Sci U S A 99:8400–8405. 12060783 10.1073/pnas.122196999 PMC123079

[CIT0109] Huggins JP Smart TS Langman S Taylor L Young T (2012) An efficient randomised, placebo-controlled clinical trial with the irreversible fatty acid amide hydrolase-1 inhibitor PF-04457845, which modulates endocannabinoids but fails to induce effective analgesia in patients with pain due to osteoarthritis of the knee. Pain 153:1837–1846. 22727500 10.1016/j.pain.2012.04.020

[CIT0110] Imbe H Kimura A Donishi T Kaneoke Y (2012) Chronic restraint stress decreases glial fibrillary acidic protein and glutamate transporter in the periaqueductal gray matter. Neuroscience 223:209–218. 22890077 10.1016/j.neuroscience.2012.08.007

[CIT0111] Ishikawa K Yasuda S Fukuhara K Iwanaga Y Ida Y Ishikawa J Yamagata H Ono M Kakeda T Ishikawa T (2014) 4-Methylcatechol prevents derangements of brain-derived neurotrophic factor and TrkB-related signaling in anterior cingulate cortex in chronic pain with depression-like behavior. Neuroreport 25:226–232. 24518228 10.1097/WNR.0000000000000072

[CIT0112] Jennings EM Okine BN Olango WM Roche M Finn DP (2015) Repeated forced swim stress differentially affects formalin-evoked nociceptive behavior and the endocannabinoid system in stress normo-responsive and stress hyper-responsive rat strains. Prog Neuropsychopharmacol Biol Psychiatry PMID: 25988529. 10.1016/j.pnpbp.2015.05.008 25988529

[CIT0113] Jennings EM Okine BN Roche M Finn DP (2014) Stress-induced hyperalgesia. Prog Neurobiol 121:1–18. 25010858 10.1016/j.pneurobio.2014.06.003

[CIT0114] Jentsch JD Andrusiak E Tran A Bowers MB Jr Roth RH (1997) Delta 9-tetrahydrocannabinol increases prefrontal cortical catecholaminergic utilization and impairs spatial working memory in the rat: blockade of dopaminergic effects with HA966. Neuropsychopharmacology 16:426–432. 9165498 10.1016/S0893-133X(97)00018-3

[CIT0115] Jesse CR Wilhelm EA Nogueira CW (2010) Depression-like behavior and mechanical allodynia are reduced by bis selenide treatment in mice with chronic constriction injury: a comparison with fluoxetine, amitriptyline, and bupropion. Psychopharmacology (Berl) 212:513–522. 20689938 10.1007/s00213-010-1977-6

[CIT0116] Ji G Fu Y Ruppert KA Neugebauer V (2007) Pain-related anxiety-like behavior requires CRF1 receptors in the amygdala. Mol Pain 3:13. 17550594 10.1186/1744-8069-3-13 PMC1891279

[CIT0117] Johnson AC Tran L Schulkin J Greenwood-Van Meerveld B (2012) Importance of stress receptor-mediated mechanisms in the amygdala on visceral pain perception in an intrinsically anxious rat. Neurogastroenterol Motil 24:479–486, e219. 22364507 10.1111/j.1365-2982.2012.01899.x PMC3461498

[CIT0118] Juhasz G Lazary J Chase D Pegg E Downey D Toth ZG Stones K Platt H Mekli K Payton A Anderson IM Deakin JF Bagdy G (2009) Variations in the cannabinoid receptor 1 gene predispose to migraine. Neurosci Lett 461:116–120. 19539700 10.1016/j.neulet.2009.06.021

[CIT0119] Kadetoff D Lampa J Westman M Andersson M Kosek E (2012) Evidence of central inflammation in fibromyalgia-increased cerebrospinal fluid interleukin-8 levels. J Neuroimmunol 242:33–38. 22126705 10.1016/j.jneuroim.2011.10.013

[CIT0120] Kappelman MD Long MD Martin C DeWalt DA Kinneer PM Chen W Lewis JD Sandler RS (2014) Evaluation of the patient-reported outcomes measurement information system in a large cohort of patients with inflammatory bowel diseases. Clin Gastroenterol Hepatol 12:1315–1323 e1312. 24183956 10.1016/j.cgh.2013.10.019 PMC4361943

[CIT0121] Katona I Rancz EA Acsady L Ledent C Mackie K Hajos N Freund TF (2001) Distribution of CB1 cannabinoid receptors in the amygdala and their role in the control of GABAergic transmission. J Neurosci 21:9506–9518. 11717385 10.1523/JNEUROSCI.21-23-09506.2001 PMC6763903

[CIT0122] Katz PS Sulzer JK Impastato RA Teng SX Rogers EK Molina PE (2015) Endocannabinoid degradation inhibition improves neurobehavioral function, blood-brain barrier integrity, and neuroinflammation following mild traumatic brain injury. J Neurotrauma 32:297–306. 25166905 10.1089/neu.2014.3508 PMC4348366

[CIT0123] Kaufmann I Hauer D Huge V Vogeser M Campolongo P Chouker A Thiel M Schelling G (2009) Enhanced anandamide plasma levels in patients with complex regional pain syndrome following traumatic injury: a preliminary report. Eur Surg Res 43:325–329. 19729930 10.1159/000235870

[CIT0124] Kendell SF Krystal JH Sanacora G (2005) GABA and glutamate systems as therapeutic targets in depression and mood disorders. Expert Opin Ther Targets 9:153–168. 15757488 10.1517/14728222.9.1.153

[CIT0125] Kerr DM Burke NN Ford GK Connor TJ Harhen B Egan LJ Finn DP Roche M (2012) Pharmacological inhibition of endocannabinoid degradation modulates the expression of inflammatory mediators in the hypothalamus following an immunological stressor. Neuroscience 204:53–63. 21952131 10.1016/j.neuroscience.2011.09.032

[CIT0126] Kerr DM Harhen B Okine BN Egan LJ Finn DP Roche M (2013) The monoacylglycerol lipase inhibitor JZL184 attenuates LPS-induced increases in cytokine expression in the rat frontal cortex and plasma: differential mechanisms of action. Br J Pharmacol 169:808–819. 23043675 10.1111/j.1476-5381.2012.02237.x PMC3687661

[CIT0127] Khongsaengdao B Louthrenoo W Srisurapanont M (2000) Depression in Thai patients with rheumatoid arthritis. J Med Assoc Thai 83:743–747. 10932508

[CIT0128] Kim H Chen L Lim G Sung B Wang S McCabe MF Rusanescu G Yang L Tian Y Mao J (2012) Brain indoleamine 2,3-dioxygenase contributes to the comorbidity of pain and depression. J Clin Invest 122:2940–2954. 22751107 10.1172/JCI61884 PMC3408737

[CIT0129] Kim J Li Y (2015) Chronic activation of CB2 cannabinoid receptors in the hippocampus increases excitatory synaptic transmission. J Physiol 593:871–886. 25504573 10.1113/jphysiol.2014.286633 PMC4398527

[CIT0130] Kirilly E Hunyady L Bagdy G (2013) Opposing local effects of endocannabinoids on the activity of noradrenergic neurons and release of noradrenaline: relevance for their role in depression and in the actions of CB(1) receptor antagonists. J Neural Transm 120:177–186. 22990678 10.1007/s00702-012-0900-1

[CIT0131] Knaster P Karlsson H Estlander AM Kalso E (2012) Psychiatric disorders as assessed with SCID in chronic pain patients: the anxiety disorders precede the onset of pain. Gen Hosp Psychiatry 34:46–52. 22001549 10.1016/j.genhosppsych.2011.09.004

[CIT0132] Knerlich-Lukoschus F Noack M von der Ropp-Brenner B Lucius R Mehdorn HM Held-Feindt J (2011) Spinal cord injuries induce changes in CB1 cannabinoid receptor and C-C chemokine expression in brain areas underlying circuitry of chronic pain conditions. J Neurotrauma 28:619–634. 21265596 10.1089/neu.2010.1652

[CIT0133] Knuth B Radtke V Rocha P da Silva KS Dalsoglio F Gazal M Jansen K Souza DO Portela LV Kaster M Oses JP (2014) Prevalence of depression symptoms and serum levels of interleukin-6 in hemodialysis patients. Psychiatry Clin Neurosci 68:275–282. 24372974 10.1111/pcn.12130

[CIT0134] Koch A Zacharowski K Boehm O Stevens M Lipfert P von Giesen HJ Wolf A Freynhagen R (2007) Nitric oxide and pro-inflammatory cytokines correlate with pain intensity in chronic pain patients. Inflamm Res 56:32–37. 17334668 10.1007/s00011-007-6088-4

[CIT0135] Koethe D Llenos IC Dulay JR Hoyer C Torrey EF Leweke FM Weis S (2007) Expression of CB1 cannabinoid receptor in the anterior cingulate cortex in schizophrenia, bipolar disorder, and major depression. J Neural Transm 114:1055–1063. 17370106 10.1007/s00702-007-0660-5

[CIT0136] Kontinen VK Kauppila T Paananen S Pertovaara A Kalso E (1999) Behavioral measures of depression and anxiety in rats with spinal nerve ligation-induced neuropathy. Pain 80:341–346. 10204747 10.1016/s0304-3959(98)00230-9

[CIT0137] Kozak KR Rowlinson SW Marnett LJ (2000) Oxygenation of the endocannabinoid, 2-arachidonylglycerol, to glyceryl prostaglandins by cyclooxygenase-2. J Biol Chem 275:33744–33749. 10931854 10.1074/jbc.M007088200

[CIT0138] Kvien TK (2004) Epidemiology and burden of illness of rheumatoid arthritis. Pharmacoeconomics 22:1–12. 15157000 10.2165/00019053-200422001-00002

[CIT0139] Kwilasz AJ Abdullah RA Poklis JL Lichtman AH Negus SS (2014) Effects of the fatty acid amide hydrolase inhibitor URB597 on pain-stimulated and pain-depressed behavior in rats. Behav Pharmacol 25:119–129. 24583930 10.1097/FBP.0000000000000023 PMC3963812

[CIT0140] Kwilasz AJ Negus SS (2012) Dissociable effects of the cannabinoid receptor agonists Delta9-tetrahydrocannabinol and CP55940 on pain-stimulated versus pain-depressed behavior in rats. J Pharmacol Exp Ther 343:389–400. 22892341 10.1124/jpet.112.197780 PMC3477211

[CIT0141] Larauche M Bradesi S Million M McLean P Tache Y Mayer EA McRoberts JA (2008) Corticotropin-releasing factor type 1 receptors mediate the visceral hyperalgesia induced by repeated psychological stress in rats. Am J Physiol Gastrointest Liver Physiol 294:G1033–1040. 18308857 10.1152/ajpgi.00507.2007

[CIT0142] La Porta C Bura AS Llorente-Onaindia J Pastor A Navarrete F Garcia-Gutierrez MS De la Torre R Manzanares J Monfort J Maldonado R (2015) Role of the endocannabinoid system in the emotional manifestations of osteoarthritis pain. Pain PMID: 26067584. 10.1097/j.pain.0000000000000260 PMC4770330 26067584

[CIT0143] Lautenbacher S Spernal J Schreiber W Krieg JC (1999) Relationship between clinical pain complaints and pain sensitivity in patients with depression and panic disorder. Psychosom Med 61:822–827. 10593634 10.1097/00006842-199911000-00015

[CIT0144] Le AM Lee M Su C Zou A Wang J (2014) AMPAkines have novel analgesic properties in rat models of persistent neuropathic and inflammatory pain. Anesthesiology 121:1080–1090. 25338127 10.1097/ALN.0000000000000351 PMC4206834

[CIT0145] Lee MC Ploner M Wiech K Bingel U Wanigasekera V Brooks J Menon DK Tracey I (2013) Amygdala activity contributes to the dissociative effect of cannabis on pain perception. Pain 154:124–134. 23273106 10.1016/j.pain.2012.09.017 PMC3549497

[CIT0146] Leggett JD Aspley S Beckett SR D’Antona AM Kendall DA (2004) Oleamide is a selective endogenous agonist of rat and human CB1 cannabinoid receptors. Br J Pharmacol 141:253–262. 14707029 10.1038/sj.bjp.0705607 PMC1574194

[CIT0147] Li JX (2015) Pain and depression comorbidity: a preclinical perspective. Behav Brain Res 276:92–98. 24797835 10.1016/j.bbr.2014.04.042 PMC4216773

[CIT0148] Lim G Sung B Ji RR Mao J (2003) Upregulation of spinal cannabinoid-1-receptors following nerve injury enhances the effects of Win 55,212-2 on neuropathic pain behaviors in rats. Pain 105:275–283. 14499445 10.1016/s0304-3959(03)00242-2

[CIT0149] Lin MC Guo HR Lu MC Livneh H Lai NS Tsai TY (2015) Increased risk of depression in patients with rheumatoid arthritis: a seven-year population-based cohort study. Clinics (Sao Paulo) 70:91–96. 25789516 10.6061/clinics/2015(02)04 PMC4351304

[CIT0150] Liu SB Zhao R Li XS Guo HJ Tian Z Zhang N Gao GD Zhao MG (2014) Attenuation of reserpine-induced pain/depression dyad by gentiopicroside through downregulation of GluN2B receptors in the amygdala of mice. Neuromolecular Med 16:350–359. 24584520 10.1007/s12017-013-8280-8

[CIT0151] Lockie SH Czyzyk TA Chaudhary N Perez-Tilve D Woods SC Oldfield BJ Statnick MA Tschop MH (2011) CNS opioid signaling separates cannabinoid receptor 1-mediated effects on body weight and mood-related behavior in mice. Endocrinology 152:3661–3667. 21810947 10.1210/en.2011-1220

[CIT0152] Lomazzo E Bindila L Remmers F Lerner R Schwitter C Hoheisel U Lutz B (2015) Therapeutic potential of inhibitors of endocannabinoid degradation for the treatment of stress-related hyperalgesia in an animal model of chronic pain. Neuropsychopharmacology 40:488–501. 25100669 10.1038/npp.2014.198 PMC4443964

[CIT0153] Lu C Liu Y Sun B Sun Y Hou B Zhang Y Ma Z Gu X (2015) Intrathecal injection of JWH-015 attenuates bone cancer pain via time-dependent modification of pro-inflammatory cytokines expression and astrocytes activity in spinal cord. Inflammation PMID: 25896633. 10.1007/s10753-015-0168-3 25896633

[CIT0154] Ludwig J Binder A Steinmann J Wasner G Baron R (2008) Cytokine expression in serum and cerebrospinal fluid in non-inflammatory polyneuropathies. J Neurol Neurosurg Psychiatry 79:1268–1273. 18550631 10.1136/jnnp.2007.134528

[CIT0155] Luo J Wang T Liang S Hu X Li W Jin F (2013) Experimental gastritis leads to anxiety- and depression-like behaviors in female but not male rats. Behav Brain Funct 9:46. 24345032 10.1186/1744-9081-9-46 PMC3878489

[CIT0156] Lutz PE Kieffer BL (2013) Opioid receptors: distinct roles in mood disorders. Trends Neurosci 36:195–206. 23219016 10.1016/j.tins.2012.11.002 PMC3594542

[CIT0157] Maciel IS Silva RB Morrone FB Calixto JB Campos MM (2013) Synergistic effects of celecoxib and bupropion in a model of chronic inflammation-related depression in mice. PLoS One 8:e77227. 24086771 10.1371/journal.pone.0077227 PMC3785450

[CIT0158] Mackie K (2008) Cannabinoid receptors: where they are and what they do. J Neuroendocrinol 20 Suppl 1:10–14. 18426493 10.1111/j.1365-2826.2008.01671.x

[CIT0159] Maes M Leonard BE Myint AM Kubera M Verkerk R (2011) The new ‘5-HT’ hypothesis of depression: cell-mediated immune activation induces indoleamine 2,3-dioxygenase, which leads to lower plasma tryptophan and an increased synthesis of detrimental tryptophan catabolites (TRYCATs), both of which contribute to the onset of depression. Prog Neuropsychopharmacol Biol Psychiatry 35:702–721. 21185346 10.1016/j.pnpbp.2010.12.017

[CIT0160] Maida V Ennis M Irani S Corbo M Dolzhykov M (2008) Adjunctive nabilone in cancer pain and symptom management: a prospective observational study using propensity scoring. J Support Oncol 6:119–124. 18402303

[CIT0161] Maletic V Raison CL (2009) Neurobiology of depression, fibromyalgia and neuropathic pain. Front Biosci (Landmark Ed) 14:5291–5338. 19482616 10.2741/3598

[CIT0162] Maneeton N Maneeton B Srisurapanont M (2013) Prevalence and predictors of pain in patients with major depressive disorder. Asian J Psychiatr 6:288–291. 23810134 10.1016/j.ajp.2012.12.004

[CIT0163] Manning BH Martin WJ Meng ID (2003) The rodent amygdala contributes to the production of cannabinoid-induced antinociception. Neuroscience 120:1157–1170. 12927220 10.1016/s0306-4522(03)00356-7

[CIT0164] Marco EM Echeverry-Alzate V Lopez-Moreno JA Gine E Penasco S Viveros MP (2014) Consequences of early life stress on the expression of endocannabinoid-related genes in the rat brain. Behav Pharmacol 25:547–556. 25083571 10.1097/FBP.0000000000000068

[CIT0165] Maric NP Adzic M (2013) Pharmacological modulation of HPA axis in depression - new avenues for potential therapeutic benefits. Psychiatr Danub 25:299–305. 24048401

[CIT0166] Mato S Vidal R Castro E Diaz A Pazos A Valdizan EM (2010) Long-term fluoxetine treatment modulates cannabinoid type 1 receptor-mediated inhibition of adenylyl cyclase in the rat prefrontal cortex through 5-hydroxytryptamine 1A receptor-dependent mechanisms. Mol Pharmacol 77:424–434. 19995940 10.1124/mol.109.060079

[CIT0167] Matsuda LA Lolait SJ Brownstein MJ Young AC Bonner TI (1990) Structure of a cannabinoid receptor and functional expression of the cloned cDNA. Nature 346:561–564. 2165569 10.1038/346561a0

[CIT0168] McLaughlin RJ Hill MN Bambico FR Stuhr KL Gobbi G Hillard CJ Gorzalka BB (2012) Prefrontal cortical anandamide signaling coordinates coping responses to stress through a serotonergic pathway. Eur Neuropsychopharmacol 22:664–671. 22325231 10.1016/j.euroneuro.2012.01.004 PMC3366159

[CIT0169] McLaughlin RJ Hill MN Dang SS Wainwright SR Galea LA Hillard CJ Gorzalka BB (2013) Upregulation of CB(1) receptor binding in the ventromedial prefrontal cortex promotes proactive stress-coping strategies following chronic stress exposure. Behav Brain Res 237:333–337. 23047058 10.1016/j.bbr.2012.09.053 PMC3501995

[CIT0170] Mechoulam R Ben-Shabat S Hanus L Ligumsky M Kaminski NE Schatz AR Gopher A Almog S Martin BR Compton DR (1995) Identification of an endogenous 2-monoglyceride, present in canine gut, that binds to cannabinoid receptors. Biochem Pharmacol 50:83–90. 7605349 10.1016/0006-2952(95)00109-d

[CIT0171] Meerwijk EL Ford JM Weiss SJ (2013) Brain regions associated with psychological pain: implications for a neural network and its relationship to physical pain. Brain Imaging Behav 7:1–14. 22660945 10.1007/s11682-012-9179-y

[CIT0172] Melis M Pistis M (2012) Hub and switches: endocannabinoid signalling in midbrain dopamine neurons. Philos Trans R Soc Lond B Biol Sci 367:3276–3285. 23108546 10.1098/rstb.2011.0383 PMC3481525

[CIT0173] Meng ID Manning BH Martin WJ Fields HL (1998) An analgesia circuit activated by cannabinoids. Nature 395:381–383. 9759727 10.1038/26481

[CIT0174] Millan MJ (2002) Descending control of pain. Prog Neurobiol 66:355–474. 12034378 10.1016/s0301-0082(02)00009-6

[CIT0175] Miller LL Picker MJ Schmidt KT Dykstra LA (2011) Effects of morphine on pain-elicited and pain-suppressed behavior in CB1 knockout and wildtype mice. Psychopharmacology (Berl) 215:455–465. 21373789 10.1007/s00213-011-2232-5 PMC3160632

[CIT0176] Miller LL Picker MJ Umberger MD Schmidt KT Dykstra LA (2012) Effects of alterations in cannabinoid signaling, alone and in combination with morphine, on pain-elicited and pain-suppressed behavior in mice. J Pharmacol Exp Ther 342:177–187. 22514333 10.1124/jpet.112.191478 PMC3383037

[CIT0177] Minocci D Massei J Martino A Milianti M Piz L Di Bello D Sbrana A Martinotti E Rossi AM Nieri P (2011) Genetic association between bipolar disorder and 524A>C (Leu133Ile) polymorphism of CNR2 gene, encoding for CB2 cannabinoid receptor. J Affect Disord 134:427–430. 21658778 10.1016/j.jad.2011.05.023

[CIT0178] Mitjans M Gasto C Catalan R Fananas L Arias B (2012) Genetic variability in the endocannabinoid system and 12-week clinical response to citalopram treatment: the role of the CNR1, CNR2 and FAAH genes. J Psychopharmacol 26:1391–1398. 22826533 10.1177/0269881112454229

[CIT0179] Mitjans M Serretti A Fabbri C Gasto C Catalan R Fananas L Arias B (2013) Screening genetic variability at the CNR1 gene in both major depression etiology and clinical response to citalopram treatment. Psychopharmacology (Berl) 227:509–519. 23407780 10.1007/s00213-013-2995-y

[CIT0180] Mitrirattanakul S Ramakul N Guerrero AV Matsuka Y Ono T Iwase H Mackie K Faull KF Spigelman I (2006) Site-specific increases in peripheral cannabinoid receptors and their endogenous ligands in a model of neuropathic pain. Pain 126:102–114. 16844297 10.1016/j.pain.2006.06.016 PMC1776167

[CIT0181] Monteleone P Bifulco M Maina G Tortorella A Gazzerro P Proto MC Di Filippo C Monteleone F Canestrelli B Buonerba G Bogetto F Maj M (2010) Investigation of CNR1 and FAAH endocannabinoid gene polymorphisms in bipolar disorder and major depression. Pharmacol Res 61:400–404. 20080186 10.1016/j.phrs.2010.01.002

[CIT0182] Moranta D Esteban S Garcia-Sevilla JA (2009) Chronic treatment and withdrawal of the cannabinoid agonist WIN 55,212-2 modulate the sensitivity of presynaptic receptors involved in the regulation of monoamine syntheses in rat brain. Naunyn Schmiedebergs Arch Pharmacol 379:61–72. 18709357 10.1007/s00210-008-0337-0

[CIT0183] Munro S Thomas KL Abu-Shaar M (1993) Molecular characterization of a peripheral receptor for cannabinoids. Nature 365:61–65. 7689702 10.1038/365061a0

[CIT0184] Naderi N Shafaghi B Khodayar MJ Zarindast MR (2005) Interaction between gamma-aminobutyric acid GABAB and cannabinoid CB1 receptors in spinal pain pathways in rat. Eur J Pharmacol 514:159–164. 15910802 10.1016/j.ejphar.2005.03.037

[CIT0185] Naderi N Haghparast A Saber-Tehrani A Rezaii N Alizadeh AM Khani A Motamedi F (2008) Interaction between cannabinoid compounds and diazepam on anxiety-like behavior of mice. Pharmacol Biochem Behav 89:64–75. 18096213 10.1016/j.pbb.2007.11.001

[CIT0186] Nagakura Y Oe T Aoki T Matsuoka N (2009) Biogenic amine depletion causes chronic muscular pain and tactile allodynia accompanied by depression: a putative animal model of fibromyalgia. Pain 146:26–33. 19646816 10.1016/j.pain.2009.05.024

[CIT0187] Narita M Kaneko C Miyoshi K Nagumo Y Kuzumaki N Nakajima M Nanjo K Matsuzawa K Yamazaki M Suzuki T (2006) Chronic pain induces anxiety with concomitant changes in opioidergic function in the amygdala. Neuropsychopharmacology 31:739–750. 16123756 10.1038/sj.npp.1300858

[CIT0188] Navarria A Tamburella A Iannotti FA Micale V Camillieri G Gozzo L Verde R Imperatore R Leggio GM Drago F Di Marzo V (2014) The dual blocker of FAAH/TRPV1 N-arachidonoylserotonin reverses the behavioral despair induced by stress in rats and modulates the HPA-axis. Pharmacol Res 87:151–159. 24861565 10.1016/j.phrs.2014.04.014

[CIT0189] Nicholl BI Mackay D Cullen B Martin DJ Ul-Haq Z Mair FS Evans J McIntosh AM Gallagher J Roberts B Deary IJ Pell JP Smith DJ (2014) Chronic multisite pain in major depression and bipolar disorder: cross-sectional study of 149,611 participants in UK Biobank. BMC Psychiatry 14:350. 25490859 10.1186/s12888-014-0350-4 PMC4297369

[CIT0190] Nogueira JB Sena LC Quintans Jde S Almeida JR Franca AV Junior LJ (2012) Side effects of the therapy with peginterferon and ribavirin in chronic hepatitis C: a small audit. J Pharm Pract 25:85–88. 21940604 10.1177/0897190011415687

[CIT0191] Norman GJ Karelina K Zhang N Walton JC Morris JS Devries AC (2010) Stress and IL-1beta contribute to the development of depressive-like behavior following peripheral nerve injury. Mol Psychiatry 15:404–414. 19773812 10.1038/mp.2009.91 PMC5214062

[CIT0192] Nunez E Benito C Pazos MR Barbachano A Fajardo O Gonzalez S Tolon RM Romero J (2004) Cannabinoid CB2 receptors are expressed by perivascular microglial cells in the human brain: an immunohistochemical study. Synapse 53:208–213. 15266552 10.1002/syn.20050

[CIT0193] O’Malley D Julio-Pieper M Gibney SM Dinan TG Cryan JF (2010) Distinct alterations in colonic morphology and physiology in two rat models of enhanced stress-induced anxiety and depression-like behavior. Stress 13:114–122. 20214436 10.3109/10253890903067418

[CIT0194] Oropeza VC Page ME Van Bockstaele EJ (2005) Systemic administration of WIN 55,212-2 increases norepinephrine release in the rat frontal cortex. Brain Res 1046:45–54. 15927549 10.1016/j.brainres.2005.03.036

[CIT0195] Osei-Hyiaman D DePetrillo M Pacher P Liu J Radaeva S Batkai S Harvey-White J Mackie K Offertaler L Wang L Kunos G (2005) Endocannabinoid activation at hepatic CB1 receptors stimulates fatty acid synthesis and contributes to diet-induced obesity. J Clin Invest 115:1298–1305. 15864349 10.1172/JCI23057 PMC1087161

[CIT0196] Overton HA Babbs AJ Doel SM Fyfe MC Gardner LS Griffin G Jackson HC Procter MJ Rasamison CM Tang-Christensen M Widdowson PS Williams GM Reynet C (2006) Deorphanization of a G protein-coupled receptor for oleoylethanolamide and its use in the discovery of small-molecule hypophagic agents. Cell Metab 3:167–175. 16517404 10.1016/j.cmet.2006.02.004

[CIT0197] Page ME Oropeza VC Sparks SE Qian Y Menko AS Van Bockstaele EJ (2007) Repeated cannabinoid administration increases indices of noradrenergic activity in rats. Pharmacol Biochem Behav 86:162–168. 17275893 10.1016/j.pbb.2006.12.020 PMC1941574

[CIT0198] Page ME Oropeza VC Van Bockstaele EJ (2008) Local administration of a cannabinoid agonist alters norepinephrine efflux in the rat frontal cortex. Neurosci Lett 431:1–5. 18055114 10.1016/j.neulet.2007.11.009 PMC2701365

[CIT0199] Palazzo E de Novellis V Petrosino S Marabese I Vita D Giordano C Di Marzo V Mangoni GS Rossi F Maione S (2006) Neuropathic pain and the endocannabinoid system in the dorsal raphe: pharmacological treatment and interactions with the serotonergic system. Eur J Neurosci 24:2011–2020. 17040473 10.1111/j.1460-9568.2006.05086.x

[CIT0200] Pare WP Redei E (1993) Depressive behavior and stress ulcer in Wistar Kyoto rats. J Physiol Paris 87:229–238. 8136789 10.1016/0928-4257(93)90010-q

[CIT0201] Parent AJ Beaudet N Beaudry H Bergeron J Berube P Drolet G Sarret P Gendron L (2012) Increased anxiety-like behaviors in rats experiencing chronic inflammatory pain. Behav Brain Res 229:160–167. 22245257 10.1016/j.bbr.2012.01.001 PMC3848972

[CIT0202] Park JM Choi MG Cho YK Lee IS Kim SW Choi KY Chung IS (2011) Cannabinoid receptor 1 gene polymorphism and irritable bowel syndrome in the Korean population: a hypothesis-generating study. J Clin Gastroenterol 45:45–49. 20505532 10.1097/MCG.0b013e3181dd1573

[CIT0203] Patel S Roelke CT Rademacher DJ Cullinan WE Hillard CJ (2004) Endocannabinoid signaling negatively modulates stress-induced activation of the hypothalamic-pituitary-adrenal axis. Endocrinology 145:5431–5438. 15331569 10.1210/en.2004-0638

[CIT0204] Pellkofer HL Havla J Hauer D Schelling G Azad SC Kuempfel T Magerl W Huge V (2013) The major brain endocannabinoid 2-AG controls neuropathic pain and mechanical hyperalgesia in patients with neuromyelitis optica. PLoS One 8:e71500. 23951176 10.1371/journal.pone.0071500 PMC3739748

[CIT0205] Pernia-Andrade AJ Kato A Witschi R Nyilas R Katona I Freund TF Watanabe M Filitz J Koppert W Schuttler J Ji G Neugebauer V Marsicano G Lutz B Vanegas H Zeilhofer HU (2009) Spinal endocannabinoids and CB1 receptors mediate C-fiber-induced heterosynaptic pain sensitization. Science 325:760–764. 19661434 10.1126/science.1171870 PMC2835775

[CIT0206] Poole H White S Blake C Murphy P Bramwell R (2009) Depression in chronic pain patients: prevalence and measurement. Pain Pract 9:173–180. 19298363 10.1111/j.1533-2500.2009.00274.x

[CIT0207] Porter AC Sauer JM Knierman MD Becker GW Berna MJ Bao J Nomikos GG Carter P Bymaster FP Leese AB Felder CC (2002) Characterization of a novel endocannabinoid, virodhamine, with antagonist activity at the CB1 receptor. J Pharmacol Exp Ther 301:1020–1024. 12023533 10.1124/jpet.301.3.1020

[CIT0208] Prescott SM Majerus PW (1983) Characterization of 1,2-diacylglycerol hydrolysis in human platelets. Demonstration of an arachidonoyl-monoacylglycerol intermediate. J Biol Chem 258:764–769. 6822511

[CIT0209] Quintero L Cardenas R Suarez-Roca H (2011) Stress-induced hyperalgesia is associated with a reduced and delayed GABA inhibitory control that enhances post-synaptic NMDA receptor activation in the spinal cord. Pain 152:1909–1922. 21636214 10.1016/j.pain.2011.04.017

[CIT0210] Racz I Nent E Erxlebe E Zimmer A (2015) CB1 receptors modulate affective behavior induced by neuropathic pain. Brain Res Bull 114:42–48. 25863168 10.1016/j.brainresbull.2015.03.005

[CIT0211] Rani Sagar D Burston JJ Woodhams SG Chapman V (2012) Dynamic changes to the endocannabinoid system in models of chronic pain. Philos Trans R Soc Lond B Biol Sci 367:3300–3311. 23108548 10.1098/rstb.2011.0390 PMC3481532

[CIT0212] Rea K Roche M Finn DP (2007) Supraspinal modulation of pain by cannabinoids: the role of GABA and glutamate. Br J Pharmacol 152:633–648. 17828292 10.1038/sj.bjp.0707440 PMC2190023

[CIT0213] Rea K Olango WM Okine BN Madasu MK McGuire IC Coyle K Harhen B Roche M Finn DP (2014) Impaired endocannabinoid signalling in the rostral ventromedial medulla underpins genotype-dependent hyper-responsivity to noxious stimuli. Pain 155:69–79. 24076311 10.1016/j.pain.2013.09.012

[CIT0214] Reich CG Mihalik GR Iskander AN Seckler JC Weiss MS (2013) Adolescent chronic mild stress alters hippocampal CB1 receptor-mediated excitatory neurotransmission and plasticity. Neuroscience 253:444–454. 24035826 10.1016/j.neuroscience.2013.08.066 PMC3827785

[CIT0215] Reich CG Taylor ME McCarthy MM (2009) Differential effects of chronic unpredictable stress on hippocampal CB1 receptors in male and female rats. Behav Brain Res 203:264–269. 19460405 10.1016/j.bbr.2009.05.013 PMC2747651

[CIT0216] Rey AA Purrio M Viveros MP Lutz B (2012) Biphasic effects of cannabinoids in anxiety responses: CB1 and GABA(B) receptors in the balance of GABAergic and glutamatergic neurotransmission. Neuropsychopharmacology 37:2624–2634. 22850737 10.1038/npp.2012.123 PMC3473327

[CIT0217] Richardson D Pearson RG Kurian N Latif ML Garle MJ Barrett DA Kendall DA Scammell BE Reeve AJ Chapman V (2008) Characterisation of the cannabinoid receptor system in synovial tissue and fluid in patients with osteoarthritis and rheumatoid arthritis. Arthritis Res Ther 10:R43. 18416822 10.1186/ar2401 PMC2453762

[CIT0218] Riebe CJ Wotjak CT (2011) Endocannabinoids and stress. Stress 14:384–397. 21663537 10.3109/10253890.2011.586753

[CIT0219] Rivat C Becker C Blugeot A Zeau B Mauborgne A Pohl M Benoliel JJ (2010) Chronic stress induces transient spinal neuroinflammation, triggering sensory hypersensitivity and long-lasting anxiety-induced hyperalgesia. Pain 150:358–368. 20573451 10.1016/j.pain.2010.05.031

[CIT0220] Roberts CJ Stuhr KL Hutz MJ Raff H Hillard CJ (2014) Endocannabinoid signaling in hypothalamic-pituitary-adrenocortical axis recovery following stress: effects of indirect agonists and comparison of male and female mice. Pharmacol Biochem Behav 117:17–24. 24316201 10.1016/j.pbb.2013.11.026 PMC3929302

[CIT0221] Roberts JD Gennings C Shih M (2006) Synergistic affective analgesic interaction between delta-9-tetrahydrocannabinol and morphine. Eur J Pharmacol 530:54–58. 16375890 10.1016/j.ejphar.2005.11.036

[CIT0222] Roche M Finn DP (2010) Brain CB2 Receptors: implications for neuropsychiatric disorders. Pharmaceuticals 3:2517–2553. 10.3390/ph3082517 PMC4033937 27713365

[CIT0223] Rock RB Gekker G Hu S Sheng WS Cabral GA Martin BR Peterson PK (2007) WIN55,212-2-mediated inhibition of HIV-1 expression in microglial cells: involvement of cannabinoid receptors. J Neuroimmune Pharmacol 2:178–183. 18040842 10.1007/s11481-006-9040-4

[CIT0224] Rodriguez JJ Mackie K Pickel VM (2001) Ultrastructural localization of the CB1 cannabinoid receptor in mu-opioid receptor patches of the rat Caudate putamen nucleus. J Neurosci 21:823–833. 11157068 10.1523/JNEUROSCI.21-03-00823.2001 PMC6762333

[CIT0225] Rodriguez-Gaztelumendi A Rojo ML Pazos A Diaz A (2014) An altered spinal serotonergic system contributes to increased thermal nociception in an animal model of depression. Exp Brain Res 232:1793–1803. 24584836 10.1007/s00221-014-3871-7

[CIT0226] Rossi S De Chiara V Musella A Sacchetti L Cantarella C Castelli M Cavasinni F Motta C Studer V Bernardi G Cravatt BF Maccarrone M Usiello A Centonze D (2010) Preservation of striatal cannabinoid CB1 receptor function correlates with the antianxiety effects of fatty acid amide hydrolase inhibition. Mol Pharmacol 78:260–268. 20424126 10.1124/mol.110.064196

[CIT0227] Rossi S Motta C Musella A Centonze D (2014) The interplay between inflammatory cytokines and the endocannabinoid system in the regulation of synaptic transmission. Neuropharmacology 96(A):105–112. 25268960 10.1016/j.neuropharm.2014.09.022

[CIT0228] Ryberg E Larsson N Sjogren S Hjorth S Hermansson NO Leonova J Elebring T Nilsson K Drmota T Greasley PJ (2007) The orphan receptor GPR55 is a novel cannabinoid receptor. Br J Pharmacol 152:1092–1101. 17876302 10.1038/sj.bjp.0707460 PMC2095107

[CIT0229] Salio C Fischer J Franzoni MF Mackie K Kaneko T Conrath M (2001) CB1-cannabinoid and mu-opioid receptor co-localization on postsynaptic target in the rat dorsal horn. Neuroreport 12:3689–3692. 11726775 10.1097/00001756-200112040-00017

[CIT0230] Scheidt CE Mueller-Becsangele J Hiller K Hartmann A Goldacker S Vaith P Waller E Lacour M (2014) Self-reported symptoms of pain and depression in primary fibromyalgia syndrome and rheumatoid arthritis. Nord J Psychiatry 68:88–92. 23586534 10.3109/08039488.2013.782566

[CIT0231] Scholich SL Hallner D Wittenberg RH Hasenbring MI Rusu AC (2012) The relationship between pain, disability, quality of life and cognitive-behavioral factors in chronic back pain. Disabil Rehabil 34:1993–2000. 22458419 10.3109/09638288.2012.667187

[CIT0232] Seif T Makriyannis A Kunos G Bonci A Hopf FW (2011) The endocannabinoid 2-arachidonoylglycerol mediates D1 and D2 receptor cooperative enhancement of rat nucleus accumbens core neuron firing. Neuroscience 193:21–33. 21821098 10.1016/j.neuroscience.2011.07.055 PMC3579619

[CIT0233] Selvarajah D Gandhi R Emery CJ Tesfaye S (2010) Randomized placebo-controlled double-blind clinical trial of cannabis-based medicinal product (Sativex) in painful diabetic neuropathy: depression is a major confounding factor. Diabetes Care 33:128–130. 19808912 10.2337/dc09-1029 PMC2797957

[CIT0234] Shahidi S Hasanein P (2011) Behavioral effects of fatty acid amide hydrolase inhibition on morphine withdrawal symptoms. Brain Res Bull 86:118–122. 21763761 10.1016/j.brainresbull.2011.06.019

[CIT0235] Shi M Wang JY Luo F (2010) Depression shows divergent effects on evoked and spontaneous pain behaviors in rats. J Pain 11:219–229. 20096641 10.1016/j.jpain.2009.07.002 PMC2835830

[CIT0236] Skrabek RQ Galimova L Ethans K Perry D (2008) Nabilone for the treatment of pain in fibromyalgia. J Pain 9:164–173. 17974490 10.1016/j.jpain.2007.09.002

[CIT0237] Smaga I Bystrowska B Gawlinski D Pomierny B Stankowicz P Filip M (2014) Antidepressants and changes in concentration of endocannabinoids and N-acylethanolamines in rat brain structures. Neurotox Res 26:190–206. 24652522 10.1007/s12640-014-9465-0 PMC4067538

[CIT0238] Smith PA Selley DE Sim-Selley LJ Welch SP (2007) Low dose combination of morphine and delta9-tetrahydrocannabinol circumvents antinociceptive tolerance and apparent desensitization of receptors. Eur J Pharmacol 571:129–137. 17603035 10.1016/j.ejphar.2007.06.001 PMC2040345

[CIT0239] Solinas M Justinova Z Goldberg SR Tanda G (2006) Anandamide administration alone and after inhibition of fatty acid amide hydrolase (FAAH) increases dopamine levels in the nucleus accumbens shell in rats. J Neurochem 98:408–419. 16805835 10.1111/j.1471-4159.2006.03880.x

[CIT0240] Song C Wang H (2011) Cytokines mediated inflammation and decreased neurogenesis in animal models of depression. Prog Neuropsychopharmacol Biol Psychiatry 35:760–768. 20600462 10.1016/j.pnpbp.2010.06.020

[CIT0241] Spitzer RL Kroenke K Linzer M Hahn SR Williams JB deGruy FV 3rd Brody D Davies M (1995) Health-related quality of life in primary care patients with mental disorders. Results from the PRIME-MD 1000 Study. JAMA 274:1511–1517. 7474219

[CIT0242] Steiner MA Marsicano G Nestler EJ Holsboer F Lutz B Wotjak CT (2008) Antidepressant-like behavioral effects of impaired cannabinoid receptor type 1 signaling coincide with exaggerated corticosterone secretion in mice. Psychoneuroendocrinology 33:54–67. 17976922 10.1016/j.psyneuen.2007.09.008 PMC2267923

[CIT0243] Suarez-Roca H Leal L Silva JA Pinerua-Shuhaibar L Quintero L (2008) Reduced GABA neurotransmission underlies hyperalgesia induced by repeated forced swimming stress. Behav Brain Res 189:159–169. 18255166 10.1016/j.bbr.2007.12.022

[CIT0244] Sugiura T Kondo S Sukagawa A Nakane S Shinoda A Itoh K Yamashita A Waku K (1995) 2-Arachidonoylglycerol: a possible endogenous cannabinoid receptor ligand in brain. Biochem Biophys Res Commun 215:89–97. 7575630 10.1006/bbrc.1995.2437

[CIT0245] Sugiura T Kondo S Sukagawa A Tonegawa T Nakane S Yamashita A Waku K (1996) Enzymatic synthesis of anandamide, an endogenous cannabinoid receptor ligand, through N-acylphosphatidylethanolamine pathway in testis: involvement of Ca(2+)-dependent transacylase and phosphodiesterase activities. Biochem Biophys Res Commun 218:113–117. 8573114 10.1006/bbrc.1996.0020

[CIT0246] Sugiura T Kondo S Kishimoto S Miyashita T Nakane S Kodaka T Suhara Y Takayama H Waku K (2000) Evidence that 2-arachidonoylglycerol but not N-palmitoylethanolamine or anandamide is the physiological ligand for the cannabinoid CB2 receptor. Comparison of the agonistic activities of various cannabinoid receptor ligands in HL-60 cells. J Biol Chem 275:605–612. 10617657 10.1074/jbc.275.1.605

[CIT0247] Sun Y Alexander SP Kendall DA Bennett AJ (2006) Cannabinoids and PPARalpha signalling. Biochem Soc Trans 34:1095–1097. 17073758 10.1042/BST0341095

[CIT0248] Suzuki T Amata M Sakaue G Nishimura S Inoue T Shibata M Mashimo T (2007) Experimental neuropathy in mice is associated with delayed behavioral changes related to anxiety and depression. Anesth Analg 104:1570–1577. 17513660 10.1213/01.ane.0000261514.19946.66

[CIT0249] Takahashi RN Ramos GA Assini FL (2003) Anxiety does not affect the antinociceptive effect of Delta 9-THC in mice: participation of cannabinoid and opioid receptors. Pharmacol Biochem Behav 75:763–768. 12957217 10.1016/s0091-3057(03)00151-5

[CIT0250] Tham SM Angus JA Tudor EM Wright CE (2005) Synergistic and additive interactions of the cannabinoid agonist CP55,940 with mu opioid receptor and alpha2-adrenoceptor agonists in acute pain models in mice. Br J Pharmacol 144:875–884. 15778704 10.1038/sj.bjp.0706045 PMC1576059

[CIT0251] Thorat SN Bhargava HN (1994) Evidence for a bidirectional cross-tolerance between morphine and delta 9-tetrahydrocannabinol in mice. Eur J Pharmacol 260:5–13. 7957626 10.1016/0014-2999(94)90003-5

[CIT0252] Thornton LM Andersen BL Blakely WP (2010) The pain, depression, and fatigue symptom cluster in advanced breast cancer: covariation with the hypothalamic-pituitary-adrenal axis and the sympathetic nervous system. Health Psychol 29:333–337. 20496988 10.1037/a0018836 PMC2910549

[CIT0253] Tsou K Brown S Sanudo-Pena MC Mackie K Walker JM (1998) Immunohistochemical distribution of cannabinoid CB1 receptors in the rat central nervous system. Neuroscience 83:393–411. 9460749 10.1016/s0306-4522(97)00436-3

[CIT0254] Tuglu C Kara SH Caliyurt O Vardar E Abay E (2003) Increased serum tumor necrosis factor-alpha levels and treatment response in major depressive disorder. Psychopharmacology (Berl) 170:429–433. 12955291 10.1007/s00213-003-1566-z

[CIT0255] Ulugol A (2014) The endocannabinoid system as a potential therapeutic target for pain modulation. Balkan Med J 31:115–120. 25207181 10.5152/balkanmedj.2014.13103 PMC4115931

[CIT0256] van der Stelt M van Kuik JA Bari M van Zadelhoff G Leeflang BR Veldink GA Finazzi-Agro A Vliegenthart JF Maccarrone M (2002) Oxygenated metabolites of anandamide and 2-arachidonoylglycerol: conformational analysis and interaction with cannabinoid receptors, membrane transporter, and fatty acid amide hydrolase. J Med Chem 45:3709–3720. 12166944 10.1021/jm020818q

[CIT0257] Van Sickle MD Duncan M Kingsley PJ Mouihate A Urbani P Mackie K Stella N Makriyannis A Piomelli D Davison JS Marnett LJ Di Marzo V Pittman QJ Patel KD Sharkey KA (2005) Identification and functional characterization of brainstem cannabinoid CB2 receptors. Science 310:329–332. 16224028 10.1126/science.1115740

[CIT0258] Vermersch P (2011) Sativex((R)) (tetrahydrocannabinol + cannabidiol), an endocannabinoid system modulator: basic features and main clinical data. Expert Rev Neurother 11:15–19. 21449855 10.1586/ern.11.27

[CIT0259] Vierck CJ Jr (2006) Mechanisms underlying development of spatially distributed chronic pain (fibromyalgia). Pain 124:242–263. 16842915 10.1016/j.pain.2006.06.001

[CIT0260] Vinod KY Xie S Psychoyos D Hungund BL Cooper TB Tejani-Butt SM (2012) Dysfunction in fatty acid amide hydrolase is associated with depressive-like behavior in Wistar Kyoto rats. PLoS One 7:e36743. 22606285 10.1371/journal.pone.0036743 PMC3351478

[CIT0261] Walczak JS Pichette V Leblond F Desbiens K Beaulieu P (2005) Behavioral, pharmacological and molecular characterization of the saphenous nerve partial ligation: a new model of neuropathic pain. Neuroscience 132:1093–1102. 15857713 10.1016/j.neuroscience.2005.02.010

[CIT0262] Walker AK Kavelaars A Heijnen CJ Dantzer R (2014) Neuroinflammation and comorbidity of pain and depression. Pharmacol Rev 66:80–101. 24335193 10.1124/pr.113.008144 PMC3880465

[CIT0263] Wang J Goffer Y Xu D Tukey DS Shamir DB Eberle SE Zou AH Blanck TJ Ziff EB (2011) A single subanesthetic dose of ketamine relieves depression-like behaviors induced by neuropathic pain in rats. Anesthesiology 115:812–821. 21934410 10.1097/ALN.0b013e31822f16ae PMC3222930

[CIT0264] Wang N Shi M Wang JY Luo F (2013) Brain-network mechanisms underlying the divergent effects of depression on spontaneous versus evoked pain in rats: a multiple single-unit study. Exp Neurol 250:165–175. 24100021 10.1016/j.expneurol.2013.09.021 PMC4386997

[CIT0265] Weber J Schley M Casutt M Gerber H Schuepfer G Rukwied R Schleinzer W Ueberall M Konrad C (2009) Tetrahydrocannabinol (delta 9-THC) Treatment in chronic central neuropathic pain and fibromyalgia patients: results of a multicenter survey. Anesthesiol Res Pract 2009: Article ID 827290. 10.1155/2009/827290 PMC2925209 20798872

[CIT0266] Wilkerson JL Gentry KR Dengler EC Wallace JA Kerwin AA Armijo LM Kuhn MN Thakur GA Makriyannis A Milligan ED (2012) Intrathecal cannabilactone CB(2)R agonist, AM1710, controls pathological pain and restores basal cytokine levels. Pain 153:1091–1106. 22425445 10.1016/j.pain.2012.02.015 PMC3603341

[CIT0267] Wilson AR Maher L Morgan MM (2008) Repeated cannabinoid injections into the rat periaqueductal gray enhance subsequent morphine antinociception. Neuropharmacology 55:1219–1225. 18723035 10.1016/j.neuropharm.2008.07.038 PMC2743428

[CIT0268] Wilson-Poe AR Morgan MM Aicher SA Hegarty DM (2012) Distribution of CB1 cannabinoid receptors and their relationship with mu-opioid receptors in the rat periaqueductal gray. Neuroscience 213:191–200. 22521830 10.1016/j.neuroscience.2012.03.038 PMC3367071

[CIT0269] Wilson-Poe AR Pocius E Herschbach M Morgan MM (2013) The periaqueductal gray contributes to bidirectional enhancement of antinociception between morphine and cannabinoids. Pharmacol Biochem Behav 103:444–449. 23063785 10.1016/j.pbb.2012.10.002 PMC3959123

[CIT0270] Wingenfeld K Nutzinger D Kauth J Hellhammer DH Lautenbacher S (2010) Salivary cortisol release and hypothalamic pituitary adrenal axis feedback sensitivity in fibromyalgia is associated with depression but not with pain. J Pain 11:1195–1202. 20627822 10.1016/j.jpain.2010.02.011

[CIT0271] Wittmann G Deli L Kallo I Hrabovszky E Watanabe M Liposits Z Fekete C (2007) Distribution of type 1 cannabinoid receptor (CB1)-immunoreactive axons in the mouse hypothalamus. J Comp Neurol 503:270–279. 17492633 10.1002/cne.21383

[CIT0272] Woolridge E Barton S Samuel J Osorio J Dougherty A Holdcroft A (2005) Cannabis use in HIV for pain and other medical symptoms. J Pain Symptom Manage 29:358–367. 15857739 10.1016/j.jpainsymman.2004.07.011

[CIT0273] Yalcin I Bohren Y Waltisperger E Sage-Ciocca D Yin JC Freund-Mercier MJ Barrot M (2011) A time-dependent history of mood disorders in a murine model of neuropathic pain. Biol Psychiatry 70:946–953. 21890110 10.1016/j.biopsych.2011.07.017

[CIT0274] Yalcin I Barthas F Barrot M (2014) Emotional consequences of neuropathic pain: insight from preclinical studies. Neurosci Biobehav Rev 47:154–164. 25148733 10.1016/j.neubiorev.2014.08.002

[CIT0275] Yasuda S Yoshida M Yamagata H Iwanaga Y Suenaga H Ishikawa K Nakano M Okuyama S Furukawa Y Furukawa S Ishikawa T (2014) Imipramine ameliorates pain-related negative emotion via induction of brain-derived neurotrophic factor. Cell Mol Neurobiol 34:1199–1208. 25156823 10.1007/s10571-014-0097-y PMC11488886

[CIT0276] Yu M Ives D Ramesha CS (1997) Synthesis of prostaglandin E2 ethanolamide from anandamide by cyclooxygenase-2. J Biol Chem 272:21181–21186. 9261124 10.1074/jbc.272.34.21181

[CIT0277] Zajkowska ZE Englund A Zunszain PA (2014) Towards a personalized treatment in depression: endocannabinoids, inflammation and stress response. Pharmacogenomics 15:687–698. 24798725 10.2217/pgs.14.40

[CIT0278] Zavitsanou K Wang H Dalton VS Nguyen V (2010) Cannabinoid administration increases 5HT1A receptor binding and mRNA expression in the hippocampus of adult but not adolescent rats. Neuroscience 169:315–324. 20438810 10.1016/j.neuroscience.2010.04.005

[CIT0279] Zeng Q Wang S Lim G Yang L Mao J Sung B Chang Y Lim JA Guo G (2008) Exacerbated mechanical allodynia in rats with depression-like behavior. Brain Res 1200:27–38. 18289511 10.1016/j.brainres.2008.01.038 PMC2386964

[CIT0280] Zhang HY Gao M Liu QR Bi GH Li X Yang HJ Gardner EL Wu J Xi ZX (2014) Cannabinoid CB2 receptors modulate midbrain dopamine neuronal activity and dopamine-related behavior in mice. Proc Natl Acad Sci U S A 111:E5007–5015. 25368177 10.1073/pnas.1413210111 PMC4246322

[CIT0281] Zhang J Hoffert C Vu HK Groblewski T Ahmad S O’Donnell D (2003) Induction of CB2 receptor expression in the rat spinal cord of neuropathic but not inflammatory chronic pain models. Eur J Neurosci 17:2750–2754. 12823482 10.1046/j.1460-9568.2003.02704.x

[CIT0282] Zhang M Dai W Liang J Chen X Hu Y Chu B Pan M Dong Z Yu S (2013) Effects of UCMS-induced depression on nociceptive behaviors induced by electrical stimulation of the dura mater. Neurosci Lett 551:1–6. 23643980 10.1016/j.neulet.2013.04.038

[CIT0283] Zhang Z Wang W Zhong P Liu SJ Long JZ Zhao L Gao HQ Cravatt BF Liu QS (2015) Blockade of 2-arachidonoylglycerol hydrolysis produces antidepressant-like effects and enhances adult hippocampal neurogenesis and synaptic plasticity. Hippocampus 25:16–26. 25131612 10.1002/hipo.22344 PMC4517601

[CIT0284] Zhao X Wang C Zhang JF Liu L Liu AM Ma Q Zhou WH Xu Y (2014) Chronic curcumin treatment normalizes depression-like behaviors in mice with mononeuropathy: involvement of supraspinal serotonergic system and GABAA receptor. Psychopharmacology (Berl) 231:2171–2187. 24297305 10.1007/s00213-013-3368-2

[CIT0285] Zhong P Wang W Pan B Liu X Zhang Z Long JZ Zhang HT Cravatt BF Liu QS (2014) Monoacylglycerol lipase inhibition blocks chronic stress-induced depressive-like behaviors via activation of mTOR signaling. Neuropsychopharmacology 39:1763–1776. 24476943 10.1038/npp.2014.24 PMC4023150

[CIT0286] Zhou W Dantzer R Budac DP Walker AK Mao-Ying QL Lee AW Heijnen CJ Kavelaars A (2015) Peripheral indoleamine 2,3-dioxygenase 1 is required for comorbid depression-like behavior but does not contribute to neuropathic pain in mice. Brain Behav Immun 46:147–153. 25637485 10.1016/j.bbi.2015.01.013 PMC4414738

[CIT0287] Zoppi S Madrigal JL Caso JR Garcia-Gutierrez MS Manzanares J Leza JC Garcia-Bueno B (2014) Regulatory role of the cannabinoid CB2 receptor in stress-induced neuroinflammation in mice. Br J Pharmacol 171:2814–2826. 24467609 10.1111/bph.12607 PMC4243857

[CIT0288] Zoppi S Perez Nievas BG Madrigal JL Manzanares J Leza JC Garcia-Bueno B (2011) Regulatory role of cannabinoid receptor 1 in stress-induced excitotoxicity and neuroinflammation. Neuropsychopharmacology 36:805–818. 21150911 10.1038/npp.2010.214 PMC3055736

[CIT0289] Zunszain PA Hepgul N Pariante CM (2013) Inflammation and depression. Curr Top Behav Neurosci 14:135–151. 22553073 10.1007/7854_2012_211

